# Stereotactic Body Radiotherapy and Liver Transplant for Liver Cancer: A Nonrandomized Controlled Trial

**DOI:** 10.1001/jamanetworkopen.2024.15998

**Published:** 2024-06-10

**Authors:** Victor Ho-Fun Lee, Varut Vardhanabhuti, Tiffany Cho-Lam Wong, Ka-On Lam, Horace Cheuk-Wai Choi, Keith Wan-Hang Chiu, Patty Pui-Ying Ho, Dennis Kwok-Chuen Leung, Matthew Ho-Man Szeto, Kwok-Fung Choi, See-Ching Chan, To-Wai Leung, Pek-Lan Khong, Chung-Mau Lo

**Affiliations:** 1 Department of Clinical Oncology, Centre of Cancer Medicine, School of Clinical Medicine, LKS Faculty of Medicine, The University of Hong Kong, Hong Kong, China; 2 Department of Clinical Oncology, Queen Mary Hospital, Hong Kong, China; 3 Department of Diagnostic Radiology, School of Clinical Medicine, LKS Faculty of Medicine, The University of Hong Kong, Hong Kong, China; 4 Department of Surgery, School of Clinical Medicine, LKS Faculty of Medicine, The University of Hong Kong, Hong Kong, China; 5 Department of Surgery, Queen Mary Hospital, Hong Kong, China; 6 Department of Diagnostic Radiology and Clinical Imaging Research Center, National University of Singapore, Singapore

## Abstract

**Importance:**

Whether stereotactic body radiotherapy (SBRT) as a bridge to liver transplant for hepatocellular carcinoma (HCC) is effective and safe is still unknown.

**Objective:**

To investigate the feasibility of SBRT before deceased donor liver transplant (DDLT) for previously untreated unresectable HCC.

**Design, Setting, and Participants:**

In this phase 2 nonrandomized controlled trial conducted between June 1, 2015, and October 18, 2019, 32 eligible patients within UCSF (University of California, San Francisco) criteria underwent dual-tracer (^18^F-fluorodeoxyglucose and ^11^C-acetate [ACC]) positron emission tomography with computed tomography (PET-CT) and magnetic resonance imaging (MRI) with gadoxetate followed by SBRT of 35 to 50 Gy in 5 fractions, and the same imaging afterward while awaiting DDLT. Statistical analysis was performed on an intention-to-treat basis between October 1 and 31, 2023.

**Intervention:**

Patients received SBRT followed by DDLT when matched deceased donor grafts were available.

**Main Outcomes and Measures:**

Coprimary end points were progression-free survival (PFS) and objective response rates (ORRs) by the Response Evaluation Criteria in Solid Tumors, version 1.1 (RECIST 1.1), modified RECIST (mRECIST), and PET Response Criteria in Solid Tumors (PERCIST). Secondary end points were local control rate, overall survival (OS), and safety.

**Results:**

A total of 32 patients (median age, 59 years [IQR, 54-63 years]; 22 men [68.8%]) with 56 lesions received SBRT. After a median follow-up of 74.6 months (IQR, 40.1-102.9 months), the median PFS was 17.6 months (95% CI, 6.6-28.6 months), and the median OS was 60.5 months (95% CI, 29.7-91.2 months). The 5-year PFS was 39.9% (95% CI, 19.9%-59.9%), and the 5-year OS was 51.3% (95% CI, 31.7%-70.9%). In terms of number of patients, ORRs were 62.5% ([n = 20] 95% CI, 54.2%-68.7%) by RECIST 1.1, 71.9% ([n = 23] 95% CI, 63.7%-79.0%) by mRECIST, and 78.1% ([n = 25] 95% CI, 73.2%-86.7%) by PERCIST. In terms of number of lesions, ORRs were 75.0% ([n = 42] 95% CI, 61.6%-80.8%) by RECIST 1.1, 83.9% ([n = 47] 95% CI, 74.7%-90.6%) by mRECIST, and 87.5% ([n = 49] 95% CI, 81.3%-98.6%) by PERCIST. Twenty patients with 36 lesions received DDLT, of whom 15 patients (75.0%) with 21 lesions (58.3%) exhibited pathologic complete response. Multivariable analyses revealed that pretreatment metabolic tumor volume (MTV) based on ACC (hazard ratio [HR], 1.06 [95% CI, 1.01-1.10]; *P* = .01) and complete metabolic response (CMR) by PERCIST (HR, 0.31 [95% CI, 0.10-0.96]; *P* = .04) were associated with PFS, while pretreatment MTV based on ACC (HR, 1.07 [95% CI, 1.03-1.16]; *P* = .01), total lesion activity based on ACC (HR, 1.01 [95% CI, 1.00-1.02]; *P* = .02), and CMR by PERCIST (HR, 0.21 [95% CI, 0.07-0.73]; *P* = .01) were associated with OS. Toxic effects associated with SBRT were reported for 9 patients (28.1%), with 1 grade 3 event.

**Conclusions and Relevance:**

This phase 2 nonrandomized controlled trial demonstrated promising survival and safety outcomes of SBRT before DDLT for unresectable HCC. Future randomized clinical trials are warranted.

## Key Points

**Question:**

Is bridging stereotactic body radiotherapy (SBRT) before deceased donor liver transplant associated with favorable outcomes among patients with unresectable hepatocellular carcinoma (HCC)?

**Findings:**

In this phase 2 nonrandomized controlled trial of 32 patients with unresectable HCC, an objective response rate of 87.5% after SBRT was observed for lesions as determined by dual-tracer positron emission tomography and magnetic resonance imaging, with significant factors associated with progression-free survival and overall survival identified. Of 20 patients who received a deceased donor liver transplant, 15 patients had pathologic complete response in the liver explants.

**Meaning:**

This study suggests that SBRT could be considered as an effective and safe bridging therapy to deceased donor liver transplant for previously untreated unresectable HCC within UCSF criteria under the surveillance of the most contemporary and accurate imaging modalities.

## Teaser

This phase 2 nonrandomized controlled trial investigates the feasibility of bridging stereotactic body radiotherapy before deceased donor liver transplant for previously untreated unresectable hepatocellular carcinoma.

## Introduction

Hepatocellular carcinoma (HCC) is a leading cause of cancer mortality in Hong Kong as well as worldwide.^1,2^ Liver transplant (LT) is among the best treatments for unresectable HCC, with appropriate patient selection, because it can also remove the premalignant cirrhotic liver. The Milan criteria and the University of California, San Francisco (UCSF) criteria provide the benchmark requirements for LT.^3,4^ However, organ shortages and long waiting times for deceased donor LT (DDLT) remain the greatest challenge. Dropout rates of 7% to 11% at 6 months and 38% at 12 months have been reported among patients waiting for DDLT.^5^ Locoregional therapies, including transarterial chemoembolization, radiofrequency ablation, radioembolization, and high-intensity focused ultrasonographic ablation, were adopted as bridging or downstaging therapy.^6,7,8^ Studies have also demonstrated comparable results with stereotactic body radiotherapy (SBRT).^8,9,10,11,12,13,14,15,16^ However, the studies were retrospective and did not use modern imaging modalities to stage HCC or evaluate objective response after SBRT.

Advanced imaging tools are essential to allow clinicians to precisely decide if patients are still within LT criteria before and after bridging or downstaging therapy and to ensure a fair listing for LT in the context of severe organ shortages. Computed tomography (CT) and magnetic resonance imaging (MRI) have been used for such purposes.^17^ Magnetic resonance imaging, especially with the liver-specific contrast agent gadoxetate disodium, further improves detection of 1- to 2-cm tumors.^18,19^ Dual-tracer (^11^C-acetate [ACC] and ^18^F-fluorodeoxyglucose [FDG]) positron emission tomography with integrated CT (PET-CT) was more sensitive and specific than FDG alone in diagnosis and surveillance.^20,21^ However, there is a paucity of data on the most appropriate imaging tool and evaluation criteria to determine objective response after SBRT for HCC. We therefore conducted a phase 2, single-arm study on SBRT as a bridging therapy to DDLT for previously untreated unresectable HCC monitored by dual-tracer PET-CT and gadoxetate-enhanced MRI. We also compared the performance of CT, MRI, and dual-tracer PET-CT in tumor objective response evaluation after SBRT.

## Methods

### Study Design and Participants

This phase 2 nonrandomized controlled trial on SBRT as bridging therapy to DDLT for patients with previously untreated unresectable HCC confirmed in a multidisciplinary tumor board, conducted between June 1, 2015, and October 18, 2019, was approved by the institutional review board of The University of Hong Kong/Hospital Authority Hong Kong West Cluster before commencement. It followed the Transparent Reporting of Evaluations With Nonrandomized Designs (TREND) reporting guideline. The full study protocol active during the time of conduct reported here is available in Supplement 1. All patients provided written informed consent. In brief, patients must fulfill UCSF LT criteria as well as the eligibility criteria for SBRT, which essentially followed the protocol^22^ of RTOG 1112 except that we adjusted the lowest limit of absolute neutrophil count to 1000 cells/µL (to convert to cells × 10^9^ per liter, multiply by 0.001), platelet count to 20 × 10^3^/µL (to convert to cells × 10^9^ per liter, multiply by 1.0), serum albumin level to 2.5 g/dL (to convert to grams per liter, multiply by 10.0), and Child-Pugh score up to B8. The acceptance criteria of SBRT plans were also slightly different from those in the protocol of RTOG 1112.^22^ Our study was registered with ClinicalTrials.gov (NCT04186234).

### Procedures

After written informed consent followed by baseline investigations with serum hematology and biochemistry, MRI, and dual-tracer PET-CT scan to confirm eligibility, all eligible patients received SBRT of 35 to 50 Gy in 5 fractions over 5 to 14 days. They were followed up weekly during SBRT, then monthly for the first 3 months, and every 3 months afterward. The 2 scans were repeated every 3 months until DDLT or radiologically documented progressive disease (PD) developed, whichever came earlier, followed by MRI or contrast-enhanced CT scans every 3 months afterward. Another course of SBRT of the same dose range and acceptance criteria could be offered to patients who developed liver-only, out-of-field recurrences while still awaiting DDLT or who developed liver-only recurrences after DDLT, provided that the interval between the first and second SBRT was 6 months or more.

Patients were prioritized for the DDLT waiting list according to the Model for End-Stage Liver Disease (MELD) scores.^23^ Since October 2009, a MELD bonus score of 18 was given to patients in our institution with T2 HCC (solitary tumor of 2-5 cm or 2-3 tumors of ≤3 cm each) who remained at T2 for 6 months or more after diagnosis.^24^ An additional 2 MELD scores were given every 3 months if the tumors remained within T2. This policy aimed to alleviate the disparity of access to DDLT between patients with HCC and those without HCC.

### Study End Points

The coprimary end points were progression-free survival (PFS, the time from SBRT to the first radiologically documented PD or death from any cause) and objective response rates (ORRs) according to the Response Evaluation Criteria in Solid Tumors, version 1.1 (RECIST 1.1); modified RECIST (mRECIST); and PET Response Criteria in Solid Tumors (PERCIST) (Supplement 1).^25^ Secondary end points were local control rate, overall survival (OS, the time from SBRT to death from any cause), and safety profiles until 6 months after SBRT or until DDLT based on the Common Terminology Criteria for Adverse Events, version 5.0.^26^ Predefined exploratory end points included factors associated with PFS and OS, factors associated with ORR, and changes in Child-Pugh scores after SBRT.

### Statistical Analysis

Statistical analysis was performed on an intention-to-treat basis between October 1 and 31, 2023. The sample size was based on the assumption that the ORR after SBRT was 85% while that after traditional bridging therapy (eg, transarterial chemoembolization or radiofrequency ablation) was 60%, reported in a retrospective study.^8^ The 2-sided type I error rate was .05, and the power was 0.8. According to the estimation by Kwak and Jung,^27^ 28 patients were required. Assuming a dropout rate of 20%, 35 patients were required for screening.

Kaplan-Meier methods and log-rank tests were performed for survival analyses and comparison between subgroups. Post hoc exploratory analyses included subgroup analyses of PFS and OS by baseline demographics performed by Cox proportional hazards regression with univariable and multivariable analyses. Changes in Child-Pugh scores after SBRT were compared by the Wilcoxon signed-rank test. Correction of significance level was not made in these exploratory analyses. The database was locked on September 30, 2023. Statistical analyses were performed by Statistical Package for Social Sciences, version 26.0 (SPSS Inc). The proportional hazard assumptions and multicollinearity of variables analyzed in Cox proportional hazards regression were tested with R software, version 4.0.4 (R Project for Statistical Computing), which verified the independence of the variables in univariable and multivariable analyses for factors associated with PFS and OS. A 2-sided *P* < .05 was considered statistically significant.

## Results

### Patient Baseline Characteristics

**Figure 1.**
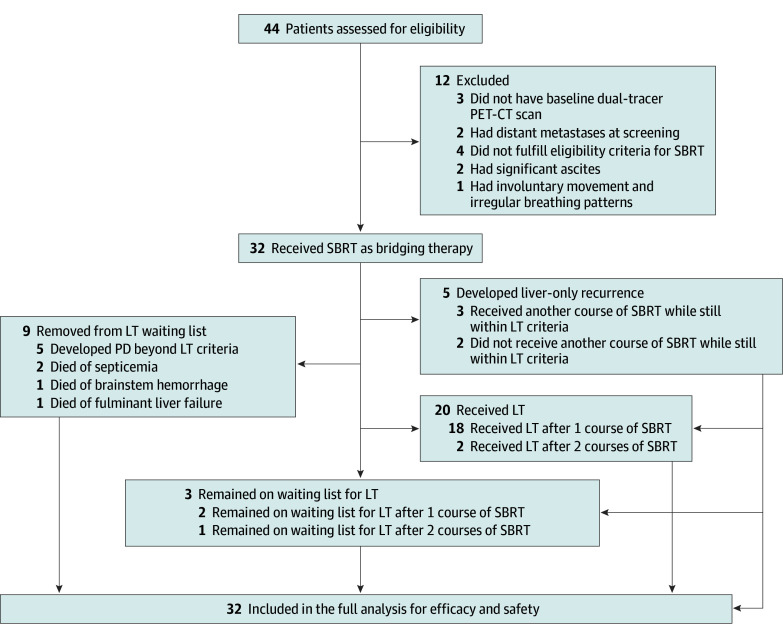
Flowchart of Trial Participants LT indicates liver transplant; PD, progressive disease; PET-CT, positron emission tomography with computed tomography; and SBRT, stereotactic body radiotherapy.

A total of 44 patients were prospectively screened for eligibility (Figure 1). Twelve patients were excluded from this study as shown. The remaining 32 patients (median age, 59 years [IQR, 54-63 years]; 22 men [68.8%] and 10 women [31.3%]) with 56 HCC lesions were eligible for SBRT. Their baseline clinical dispositions are shown in Table 1.

**Table 1.** Patient Baseline Characteristics

Characteristic	**Patients, No. (%) (N = 32)**
Age, median (IQR), y	59 (54-63)
Sex	
Female	10 (31.3)
Male	22 (68.8)
Child-Pugh score	
5	8 (25.0)
6	12 (37.5)
7	6 (18.8)
8	6 (18.8)
ALBI grade, median (IQR)	−2.2 (−2.6 to 1.8)
HALT-HCC score, median (IQR)	12.1 (10.7 to 15.1)
<17	28 (87.5)
≥17	4 (12.5)
Hepatitis B carrier	28 (87.5)
Hepatitis C carrier	4 (12.5)
Nonhepatitis B or C	0
Baseline platelet count, median (IQR), ×10^3^/µL	52 (34 to 83)
Baseline serum albumin, median (IQR), g/dL	3.7 (3.3 to 4.1)
Baseline serum bilirubin, median (IQR), mg/dL	1.6 (1.1 to 2.1)
Baseline international normalized ratio, median (IQR)	1.3 (1.2 to 1.4)
Baseline serum α-fetoprotein, median (IQR), ng/mL	10.5 (5.3 to 37.0)
Ascites present before SBRT	
None	31 (96.9)
Mild	1 (3.1)
Moderate to severe	0
Hepatic encephalopathy present before SBRT	0
MELD score, median (IQR)	12.3 (9.6 to 15.0)
MELD-Na score, median (IQR)	11.3 (7.1 to 14.0)
Tumor burden, median (IQR)^a^	5.1 (4.1 to 6.2)
Tumor burden score, median (IQR)^b^	3.9 (3.2 to 4.6)
No. of HCC lesions	
1	17 (53.1)
2	7 (21.9)
3	8 (25.0)
Segment where HCC lesion(s) were located (56 lesions total)	
1	1 (1.8)
2	4 (7.1)
3	2 (3.6)
4	6 (10.7)
5	7 (12.5)
6	13 (23.2)
7	7 (12.5)
8	16 (28.6)
Met Milan criteria	
Yes	22 (68.8)
No	10 (31.3)
Met UCSF criteria	
Yes	32 (100)
No	0
Met up-to-7 criteria	
Yes	32 (100)
No	0
Baseline ACC SUVmax, median (IQR)	3.8 (2.5 to 6.2)
Baseline MTV based on ACC uptake, median (IQR)	3.5 (1.3 to 6.9)
Baseline total lesion activity based on ACC uptake, median (IQR)	9.5 (3.5 to 30.5)
Baseline FDG SUVmax, median (IQR)	2.1 (2.1 to 2.2)
Baseline MTV based on FDG, median (IQR)	0.9 (0.0 to 1.6)
Baseline total lesion glycolysis based on FDG uptake, median (IQR)	1.7 (0.0 to 2.5)
Baseline largest diameter of HCC lesions in centimeters based on RECIST 1.1, median (IQR)	2.6 (2.1 to 3.4)
Baseline largest diameter of HCC lesions in centimeters based on mRECIST, median (IQR)	2.6 (2.1 to 3.3)
Gross tumor volume, median (IQR), mL	10.0 (4.0 to 28.4)
Planning tumor volume, median (IQR), mL	25.5 (16.7 to 62.7)
Dose of SBRT (Gy in 5 fractions) (56 lesions)	
35	3 (5.4)
38	3 (5.4)
40	3 (5.4)
43	3 (5.4)
45	19 (33.9)
50	25 (44.6)

Abbreviations: ACC, ^11^C-acetate; ALBI, albumin-bilirubin grade; FDG, ^18^F-fluorodeoxyglucose; HALT-HCC, Hazard Associated with Liver Transplantation for Hepatocellular Carcinoma; HCC, hepatocellular carcinoma; MELD, Model for End-Stage Liver Disease; MELD-Na, MELD sodium; mRECIST, Modified Response Evaluation Criteria in Solid Tumors; MTV, metabolic tumor volume; RECIST 1.1, Response Evaluation Criteria in Solid Tumors, version 1.1; SBRT, stereotactic body radiotherapy; SUVmax, maximum standardized uptake value; UCSF, University of California, San Francisco.

SI conversion factors: To convert platelet count to cells × 10^9^ per liter, multiply by 1.0, albumin to grams per liter, multiply by 10.0; bilirubin to micromoles per liter, multiply by 17.104; and α-fetoprotein to micrograms per liter, multiply by 1.0.

^a^ Calculated as the sum of the number of nodules and the size (in centimeters) of the largest nodule.

^b^ Calculated as the square root of sum of the squares of the maximum tumor diameter and the squares of the number of liver lesions.

The median maximum standardized uptake value (SUVmax) of FDG and ACC of the normal unaffected liver was 2.1 (IQR, 2.0-2.2). In general, the tumors took up more ACC (median SUVmax, 3.8 [IQR, 2.0-12.6]) than FDG (median SUVmax, 2.1 [IQR, 2.0-7.6]) (*P* < .001). One small tumor of 0.3 cm^3^ was seen only on MRI scan but not on PET-CT or contrast-enhanced CT scans. On the other hand, dual-tracer PET-CT scans with ACC and MRI scans detected 4 (7.1%) additional HCC lesions that were inconspicuous in contrast-enhanced CT scans.

The metabolic tumor volume (MTV) and total lesion glycolysis based on FDG or the MTV and total lesion activity based on ACC were also captured and compared (methods of determining MTV, total lesion glycolysis, and total lesion activity are described in Supplement 1). The MTV and total lesion activity based on ACC could be determined in 31 patients (96.9%) and 55 lesions (98.2%), compared with 6 patients (18.8%) and 9 lesions (16.1%) based on FDG (*P* < .001). The MTV and total lesion activity based on ACC were significantly larger than the MTV and total lesion glycolysis based on FDG.

The median radiation dose was 45 Gy (IQR, 35-50 Gy). Seventeen patients (53.1%) with 25 lesions (44.6%) received a full dose of 50 Gy. Two patients (6.3%) with 3 HCC lesions (5.4%) received 35 Gy because their tumors abutted the stomach and the duodenum, which limited the maximum radiation dose allowed. The radiation dosimetric parameters of the target volumes and the important organs at risk are shown in eTable 1 and eTable 2 in Supplement 2. Three patients (9.4%) received another SBRT at 10.5, 12.0, and 16.7 months for newly developed solitary and out-of-field liver-only recurrences after their first SBRT while they were still within LT criteria, of whom 2 subsequently received DDLT 2 weeks and 2.4 months, respectively, after their second course of SBRT; 1 patient was still on the waiting list for DDLT at the time of publication. These 2 patients, with lesions treated with a second course of SBRT shortly followed by DDLT without any post-SBRT imaging, were excluded from statistical analyses.

mRECIST showed moderately good agreement with RECIST 1.1 (κ = 0.640; *P* < .001) but weak agreement with PERCIST (κ = 0.243; *P* = .01). On the other hand, PERCIST showed weak agreement with RECIST 1.1 (κ = 0.192; *P* = .02). When evaluating ORRs by RECIST 1.1, 20 patients (62.5% [95% CI, 54.2%-68.7%]) and 42 lesions (75.0% [95% CI, 61.6%-80.8%]) demonstrated an objective response, in which 8 patients (25.0%) and 21 lesions (37.5%) had complete response (CR). On the other hand, with mRECIST as the evaluation criteria, objective response was observed for 23 patients (71.9% [95% CI, 63.7%-79.0%]) and 47 lesions (83.9% [95% CI, 74.7%-90.6%]), in which 14 patients (43.8%) and 33 lesions (58.9%) had CR. Objective metabolic response (including complete metabolic response [CMR] and partial metabolic response with respect to ACC uptake) with PERCIST as the evaluation criteria was seen for 25 patients (78.1% [95% CI, 73.2%-86.7%]) and 49 lesions (87.5% [95% CI, 81.3%-98.6%]), in which 19 patients (59.4%) and 40 lesions (71.4%) demonstrated CMR. A case showing CMR is illustrated in eFigure 1 in Supplement 2. Overall, the local control defined by RECIST 1.1, mRECIST, and PERCIST was 100%.

Stereotactic body radiotherapy doses of more than 35 Gy were associated with at least partial response defined by mRECIST and at least partial metabolic response by PERCIST, and marginally associated with at least partial response by RECIST 1.1. Twenty patients (62.5%) with 36 tumors received DDLT, including the 2 aforementioned patients who received 2 courses of SBRT. The median interval between SBRT and DDLT was 6.9 months (IQR, 1.5-19.4 months). Among these 20 patients and 36 tumors, pathologic complete response was demonstrated in 21 lesions (58.3%) in 15 patients (eFigure 2 in Supplement 2). Another 12 of these lesions (33.3%) in 10 patients had major pathologic response with more than 90% necrosis. Only 3 of these HCC lesions (8.3%) in 3 patients exhibited less than 50% necrosis.

One patient developed a new HCC in the liver explant that was not seen radiologically before DDLT, when he waited 4 months after SBRT before DDLT, albeit a pathologic complete response was observed in all SBRT-treated HCC lesions. Another patient had 4 newly developed HCCs less than 5 mm in the liver explant despite a pathologic complete response and stable disease in his 2 SBRT-treated lesions. Another patient had persistent HCC in 1 of 2 SBRT-treated lesions, while the other SBRT-treated lesion demonstrated a pathologic complete response. Last, 2 of the newly developed solitary HCC lesions in the previously mentioned 2 patients who received their second SBRT shortly followed by DDLT remained the same in size in the explants. Taking the pathologic analysis as the reference standard, the sensitivity of detection of HCC was higher with MRI (87.2% [34 of 39]) and dual-tracer PET-CT (87.2% [34 of 39]) compared with contrast-enhanced CT (79.5% [31 of 39]).

Four patients (20.0%) developed PD (at 3.6, 7.5, 12.4, and 24.1 months, respectively) after DDLT, including bone metastases in the first 2 patients with earlier relapses and liver-only, out-of-field recurrences as late relapses in the remaining 2 patients, who subsequently received another course of SBRT. Three of 20 patients (15.0%) died after DDLT, including the 2 patients who developed bone metastases as mentioned and another patient who died of pneumonia 3.4 years after DDLT. Their median PFS was 12.7 months (95% CI, 4.7-20.6 months) calculated from the starting date of SBRT and 7.5 months (95% CI, 0.0-16.1 months) calculated from the date of DDLT, respectively, and their median OS was 19.9 months (95% CI, 0.1-39.7 months) calculated from the starting date of SBRT and 14.0 months (95% CI, 0.00-33.7 months) calculated from the date of DDLT, respectively.

Nine patients were removed from the LT waiting list after SBRT. Four patients died, including 1 from sudden brainstem hemorrhage (2 months after SBRT), 2 secondary to septicemia (5 and 8 months, respectively, after SBRT), and 1 from fulminant liver failure secondary to progressive cirrhosis without residual HCC (22.4 months after SBRT). The remaining 5 patients developed multifocal intrahepatic PD that was beyond LT criteria. The LT dropout rate secondary to HCC progression after SBRT was 15.6%. Three patients were still on the waiting list for DDLT.

### Survival Outcomes

**Figure 2.**
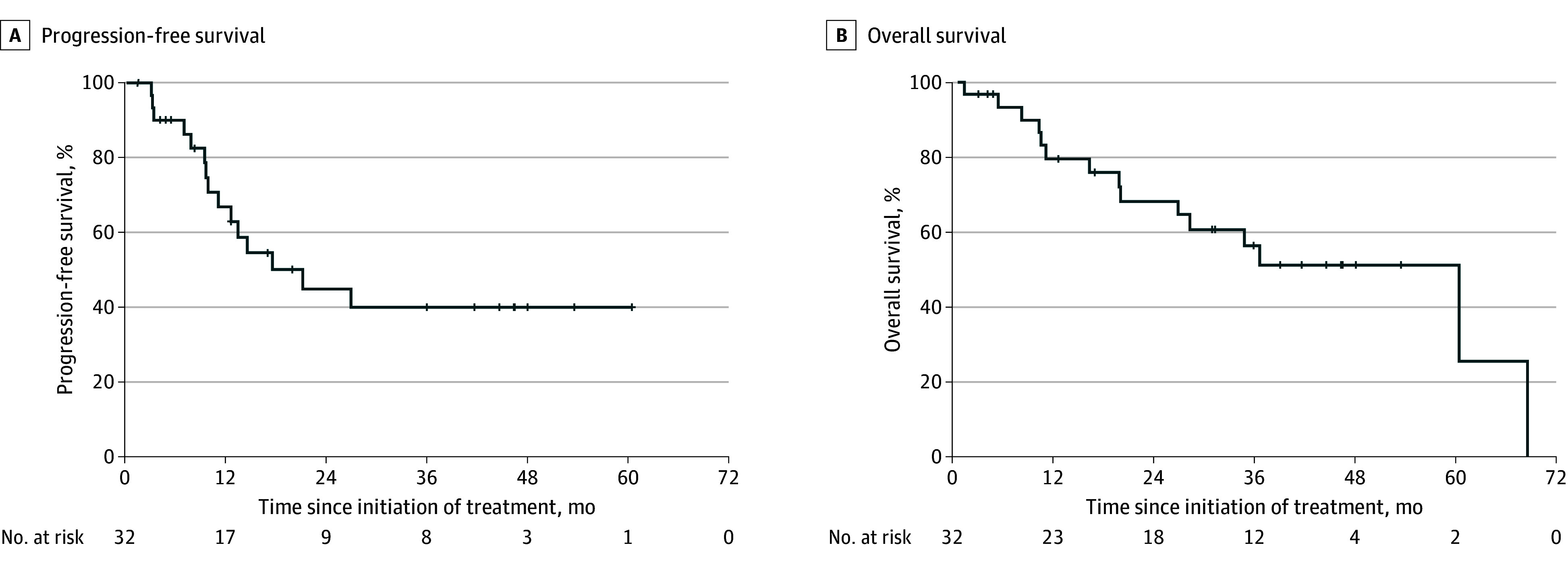
Progression-Free Survival and Overall Survival Kaplan-Meier estimations show progression-free survival (A) and overall survival (B) of the whole study population. Crosses indicate censoring.

After a median follow-up of 74.6 months (IQR, 40.1-102.9 months), 17 patients (53.1%) were still alive and free from PD. Of the 11 patients who died, 8 died without receiving DDLT (1 died of brainstem hemorrhage, 2 died of septicemia, 1 died of fulminant liver failure secondary to progressive cirrhosis without residual HCC, and 4 died of HCC progression after SBRT who were subsequently removed from the DDLT waiting list, as described before), and 3 died after DDLT (2 died of HCC with bone metastases and 1 died of pneumonia, as mentioned). Fourteen patients (43.8%) developed PD: 5 patients (35.7%) had intrahepatic PD beyond LT criteria, 2 patients developed liver-only recurrence and received second SBRT followed by DDLT, 1 patient developed liver-only recurrence treated with second SBRT while still awaiting DDLT, 2 patients had liver-only recurrence after SBRT and soon received LT before considering second SBRT, and 4 patients developed PD after DDLT, as previously mentioned. The median PFS was 17.6 months (95% CI, 6.6-28.6 months) with a 5-year PFS rate of 39.9% (95% CI, 19.9%-59.9%) (Figure 2A). The median OS was 60.5 months (95% CI, 29.7-91.2 months), with a 5-year OS rate of 51.3% (95% CI, 31.7%-70.9%) (Figure 2B).

Patients with CMR in all SBRT-treated lesions had a longer median PFS (not reached; *P* = .03) and OS (60.5 months; *P* = .009) compared with those whose tumors did not attain CMR (median PFS, 10.0 months; median OS, 19.9 months) (eFigure 3 in Supplement 2). Patients whose lesions were within the Milan criteria had a trend of longer PFS (median, 27.0 vs 10.0 months; *P* = .08) and a significantly longer OS (median, 60.5 vs 19.9 months; *P* = .01) (eFigure 4 in Supplement 2). Those who received DDLT had a significantly longer PFS (median, not reached vs 10.0 months; *P* = .008) and OS (median, not reached vs 11.2 months; *P* < .001) (eFigure 5 in Supplement 2).

In multivariable analysis, pretreatment MTV based on ACC (hazard ratio [HR], 1.06 [95% CI, 1.01-1.10]; *P* = .01), and CMR by PERCIST (HR, 0.31 [95% CI, 0.10-0.96]; *P* = .04) were associated with PFS (Table 2), while pretreatment MTV (HR, 1.07 [95% CI, 1.03-1.16]; *P* = .01) and total lesion activity based on ACC uptake (HR, 1.01 [95% CI, 1.00-1.02]; *P* = .02), CR by mRECIST (HR, 0.24 [95% CI, 0.06-0.95]; *P* = .04), and CMR by PERCIST (HR, 0.21 [95% CI, 0.07-0.73]; *P* = .01) were associated with OS (Table 3). Stereotactic body radiotherapy was generally well tolerated and manageable. Eight patients (25.0%) developed grade 1 elevated serum liver transaminases that subsided within 3 months of SBRT. Three patients (9.4%) had grade 1 dyspepsia that subsided spontaneously within 1 month of SBRT. Only 1 case of grade 3 ascites was observed in 1 patient, which returned to baseline after drainage. A transient increase in Child-Pugh scores was noted for 13 patients (40.6%), which became significant at 3 months but insignificant at 6 months after SBRT (eTables 3 and 4 in Supplement 2). No patients had a decrease in platelet level to 10 × 10^3^/µL or less, developed hemorrhage, or required platelet transfusion.

**Table 2.** Univariable and Multivariable Analyses for Progression-Free Survival

Covariates	Univariable analysis		Multivariable analysis	
	Hazard ratio (95% CI)	*P* value^a^	Hazard ratio (95% CI)	*P* value^a^
Age	1.10 (1.00-1.20)	.06	1.10 (1.00-1.15)	.08
Pretreatment Child-Pugh score	0.81 (0.47-1.25)	.47	NA	NA
Pretreatment ALBI grade	0.68 (0.23-2.01)	.48	NA	NA
Pretreatment HALT-HCC score	1.06 (0.95-1.19)	.31	NA	NA
Pretreatment tumor burden score	1.30 (0.77-2.19)	.33	NA	NA
Pretreatment AFP	1.00 (1.00-1.00)	.12	NA	NA
Pretreatment SUVmax of FDG	1.04 (0.68-1.59)	.85	NA	NA
Pretreatment SUVmax of ACC	0.98 (0.84-1.14)	.77	NA	NA
Pretreatment MTV based on FDG uptake^b^	1.01 (0.95-1.11)	.82	NA	NA
Pretreatment TLG based on FDG uptake^b^	1.00 (0.98-1.20)	.91	NA	NA
Pretreatment MTV based on ACC uptake^b^	1.06 (1.01-1.10)	.02	1.06 (1.01-1.10)	.01
Pretreatment TLA based ACC uptake	1.01 (1.00-1.02)	.09	1.00 (1.00-1.03)	.16
Total gross tumor volumes	1.00 (0.99-1.03)	.22	NA	NA
Total planning target volume	1.01 (1.00-1.02)	.30	NA	NA
Complete response by RECIST 1.1	0.86 (0.27-2.70)	.79	NA	NA
Complete response by mRECIST	0.45 (0.15-0.32)	.15	NA	NA
Complete metabolic response by PERCIST	0.33 (0.12-0.92)	.03	0.31 (0.10-0.96)	.04

Abbreviations: ACC, ^11^C-acetate; AFP, α-fetoprotein; ALBI, albumin-bilirubin grade; FDG, ^18^F-fluorodeoxyglucose; HALT-HCC, Hazard Associated with Liver Transplantation for Hepatocellular Carcinoma; mRECIST, Modified Response Evaluation Criteria in Solid Tumors; MTV, metabolic tumor volume; NA, not applicable; PERCIST, Positron Emission Tomography Response Criteria in Solid Tumors; RECIST 1.1, Response Evaluation Criteria in Solid Tumors, version 1.1; SUVmax, maximum standardized uptake value; TLA, total lesion activity; TLG, total lesion glycolysis.

^a^ Only covariates found significant in univariable analysis (*P* < .10) will be considered in multivariable analysis.

^b^ The method of determination of MTV and TLG was described in the protocol (Supplement 1).

**Table 3.** Univariable and Multivariable Analyses for Overall Survival

Covariates	Univariable analysis		Multivariable analysis	
	Hazard ratio (95% CI)	*P* value^a^	Hazard ratio (95% CI)	*P* value^a^
Age	1.04 (0.94-1.14)	.47	NA	NA
Pretreatment Child-Pugh score	0.84 (0.48-1.48)	.54	NA	NA
Pretreatment ALBI grade	0.73 (0.25-2.08)	.55	NA	NA
Pretreatment HALT-HCC score	1.03 (0.92-1.17)	.60	NA	NA
Pretreatment tumor burden score	1.48 (0.84-2.62)	.18	NA	NA
Pretreatment AFP	1.00 (1.00-1.00)	.94	NA	NA
Pretreatment SUVmax of FDG	0.57 (0.12-2.61)	.46	NA	NA
Pretreatment SUVmax of ACC	0.91 (0.70-1.19)	.50	NA	NA
Pretreatment MTV based on FDG uptake^b^	1.02 (0.99-1.10)	.91	NA	NA
Pretreatment TLG based on FDG uptake^b^	1.01 (0.94-1.10)	.88	NA	NA
Pretreatment MTV based on ACC uptake^b^	1.08 (1.02-1.14)	.04	1.07 (1.03-1.16)	.01
Pretreatment TLA based on ACC uptake	1.01 (1.00-1.02)	.02	1.01 (1.00-1.02)	.02
Total gross tumor volumes	1.02 (1.00-1.04)	.06	1.00 (1.00-1.04)	.05
Total planning target volume	1.01 (1.00-1.02)	.22	NA	NA
Complete response by RECIST 1.1	0.62 (0.17-2.27)	.47	NA	NA
Complete response by mRECIST	0.26 (0.07-0.96)	.04	0.24 (0.06-0.95)	.04
Complete metabolic response by PERCIST	0.23 (0.07-0.75)	.01	0.21 (0.07-0.73)	.01

Abbreviations: ACC, ^11^C-acetate; AFP, α-fetoprotein; ALBI, albumin-bilirubin grade; FDG, ^18^F-fluorodeoxyglucose; HALT-HCC, Hazard Associated with Liver Transplantation for Hepatocellular Carcinoma; mRECIST, Modified Response Evaluation Criteria in Solid Tumors; MTV, metabolic tumor volume; NA, not applicable; PERCIST, Positron Emission Tomography Response Criteria in Solid Tumors; RECIST 1.1, Response Evaluation Criteria in Solid Tumors, version 1.1; SUVmax, maximum standardized uptake value; TLA, total lesion activity; TLG, total lesion glycolysis.

^a^ Only covariates found significant in univariable analysis (*P* < .10) will be considered in multivariable analysis.

^b^ The method of determination of MTV and TLG was described in the protocol (Supplement 1).

## Discussion

There are several important highlights in this study. First, to our knowledge, this is the first prospective study on SBRT as a bridge to DDLT with tumor response gauged by the most contemporaneous imaging tools. Second, to our knowledge, this was the first study using dual-tracer PET-CT in HCC diagnosis and staging, ORR evaluation, and surveillance after SBRT. Third, to our knowedge, this is the first study using PERCIST based on ACC to evaluate ORR.^25^ Fourth, we identified that both the pretreatment volumetric parameters (gross tumor volume, MTV, and total lesion activity) and the objective response status were independent factors associated with PFS and OS. Fifth, we adopted a more lenient and realistic set of eligibility criteria for SBRT and acceptance criteria for radiotherapy treatment plans.

A more lenient set of eligibility criteria (accepting a Child-Pugh score up to B8, lower serum albumin level, and lower platelet count) compared with the XXL (Liver Transplantation in Hepatocellular Carcinoma After Tumour Downstaging) study^28^ and higher dose constraints allowing for certain organs at risk close to the liver compared with the RTOG 1112 protocol and other international guidelines were used in our study,^22,28,29^ as Asian patients with hepatitis B usually have more shrunken cirrhotic livers, resulting in lower serum albumin levels and very low platelet counts, precluding the option of other locoregional therapies. Otherwise, 23 patients (71.9%) would have not been eligible for our study, and the SBRT plans of another 7 patients (21.9%) would have been be deemed unacceptable if we had adhered to the original eligibility and SBRT acceptance criteria of the RTOG 1112 trial (eTables 5 and 6 in Supplement 2). A very low incidence of SBRT-related hepatotoxic effects was observed in our study.

We allowed a higher minimum radiation dose of 35 Gy or more to the tumors instead of 27.5 Gy used in the RTOG 1112 trial. We are aware that some patients never received LT because of a serious paucity of organs, small liver graft volume, and the overarching principles of organ donation and allocation of each country or region, regardless of the objective response after bridging therapy. Therefore, we delivered the highest possible radiation dose to achieve more durable disease control, which may allow patients to wait longer for DDLT. It would be unethical to intentionally deliver a lower radiation dose and produce a suboptimal response so that patients would be placed as higher priorities for DDLT, or to not transplant a liver to a patient who had CR after SBRT when he or she was at the top of the DDLT waitlist or there was no other suitable recipient.

### Limitations

We acknowledge the following study limitations. Our study is not designed to compare the sensitivity and specificity of dual-tracer PET-CT with the sensitivity and specificity of MRI. Further investigations into correlation of the most optimal dose fractionation of SBRT with treatment response, predictive biomarkers of pathologic response in the liver explants, and potential surrogate end points should also be addressed in larger phase 3 trials. Moreover, one may argue that competing risk analysis is the ideal method to evaluate survival in studies on organ transplant, which is considered a censoring event.^30^ An organ transplant followed by lifelong immunosuppressive therapy may have altered the risk of events among patients who may have developed cancer after a kidney transplant for their chronic kidney disease. In the context of LT for HCC, competing risk analyses are most commonly applied in retrospective studies in which all the tumor-related or nontumor-related events have occurred^31^ and are less appropriate in prospective studies in which nontumor-related events may not occur at all, as best illustrated in the recent XXL study in which competing life analyses were not considered in survival analysis.^28^ Competing risk analysis is less appropriate for our study, in which the most predominant event after LT was HCC progression and HCC-related death, commonly occurring among Asian patients whose hepatitis B infection was reactivated as a result of prolonged immunosuppressive therapy even though maximal antiviral therapy was given. Only 1 of 20 DDLT recipients died of a nontumor event with pneumonia in our study.

## Conclusions

This nonrandomized controlled trial demonstrates the efficacy and safety of SBRT as a bridging therapy before DDLT for previously untreated unresectable HCC. We also demonstrated the feasibility and reliability of dual-tracer PET-CT and gadoxetate-enhanced MRI in tumor response evaluation.

## Notes

Supplement 1.
Trial Protocol

Supplement 2. **eFigure 1.** Dual-Tracer PET-CT Images With Dual Tracers (11C-Acetate [ACC] and 18F-Deoxyglucose [FDG]) in a 60-Year-Old Patient With Two Hepatocellular Carcinoma Lesions in (A) Segment 4b (SUVmax 3.6 With ACC) and (B) Segment 6 (SUVmax 2.9 With ACC) Before (Indicated by Arrows) and After Stereotactic Body Radiation Therapy (SBRT) **eFigure 2.** Formalin-Fixed Paraffin-Embedded Sections Under Microscopy in (A) Low-Power Field, and (B) High-Power Field Demonstrating Pathological Complete Response of a Hepatocellular Carcinoma Organized by a Fibrous Capsule in the Background of Cirrhosis Following Stereotactic Body Radiation Therapy **eFigure 3.** (A) Progression-Free Survival of Patients Who Had Complete Metabolic Response (CMR) and Those Who Did Not Have CMR Defined by PERCIST After Stereotactic Body Radiation Therapy and (B) Overall Survival of Patients Who Had Complete Metabolic Response (CMR) and Those Who Did Not Have CMR Defined by PERCIST After Stereotactic Body Radiation Therapy **eFigure 4.** (A) Progression-Free Survival of Patients Whose Hepatocellular Carcinoma Were Within the Milan Criteria and Beyond the Milan Criteria and (B) Overall Survival of Patients Whose Hepatocellular Carcinoma Were Within the Milan Criteria and Beyond the Milan Critieria **eFigure 5.** (A) Progression-Free Survival of Patients Who Received and Did Not Receive Liver Transplantation After Stereotactic Body Radiation Therapy and (B) Overall Survival of Patients Who Received and Did Not Receive Liver Transplantation After Stereotactic Body Radiation Therapy **eTable 1.** Dose Parameters of Gross Tumor Volumes and Planning Target Volumes of All Hepatocellular Carcinoma Lesions Treated With Stereotactic Body Radiation Therapy **eTable 2.** Dose Parameters of All Important Organs-at-Risk of All Patients **eTable 3.** Child-Pugh Scores Before and After Stereotactic Body Radiation Therapy (N = 32) **eTable 4.** Change in Child-Pugh Scores After Stereotactic Body Radiation Therapy (N = 32) **eTable 5.** Number of Patients Whose Pretreatment Parameters Are Beyond RTOG 1112 Trial Eligibility Criteria **eTable 6.** Number of Patients Whose Dosimetric Parameters of Organs-at-Risk in Their SBRT Plans Beyond RTOG 1112 Trial Acceptance Criteria

Supplement 3.
Data Sharing Statement
