# Supplementary material for: Stereotactic Body Radiotherapy and Liver Transplant for Liver Cancer: A Nonrandomized Controlled Trial
Source: JAMA Netw Open. 2024 Jun 10;7(6):e2415998. doi: 10.1001/jamanetworkopen.2024.15998 (PMC11165380; doi:10.1001/jamanetworkopen.2024.15998)
Supplement: Supplement 1. — Trial Protocol [file jamanetwopen-e2415998-s001.pdf]

**Protocol Title:**	FDG and 11C-acetate PET-CT scan and magnetic resonance imaging with gadoxetate disodium to assess tumor response after stereotactic body radiation therapy as bridging therapy for patients with hepatocellular carcinoma awaiting liver transplantation *
**Phase:**	2
**Protocol Publication Date:**	1 June 2015
**Sponsor:**	Department of Clinical Oncology, LKS Faculty of Medicine, The University of Hong Kong
**Principal Investigators:**	Dr. Victor Ho Fun LEE<br><br>Department of Clinical Oncology<br><br>LKS Faculty of Medicine<br><br>The University of Hong Kong<br><br>Email: vhflee@hku.hk

PROTOCOL SYNOPSIS

**Title**	FDG and 11C-acetate PET-CT scan and magnetic resonance imaging with gadoxetate disodium to assess tumor response after stereotactic body radiation therapy as bridging therapy for patients with hepatocellular carcinoma awaiting liver transplantation *
**Phase**	II
**Rationale**	Hepatocellular carcinoma (HCC) is one of the commonest solid malignancies in Asia Pacific region, primarily because of high incidence of hepatitis B infection in this locality.<br><br>Liver transplantation (LT) is the curative treatment of choice for HCC as it has the advantage of removing the tumor and also the premalignant cirrhotic liver. Milan (solitary tumor < 5cm, or up to 3 tumors, each < 3cm) and University of California, San Francisco (UCSF) criteria (solitary tumor ≤ 6.5cm, up to 3 tumors with none > 4.5cm, and total tumor diameter ≤ 8cm) provide the benchmark requirements for LT, at which a 5-year survival of > 70% and recurrence rate ranging from 5 to 15% can be achieved. However, organ shortage and waiting time for liver grafts remain the greatest challenge. The dropout rate of LT was reported to range from 10% to 40%, depending on the organ availabilities in different countriese/regions. Several locoregional treatment including transarterial chemoembolization (TACE), radiofrequency ablation (RFA), yttrium-90 transarterial radioembolization (TARE), and high-intensity focused ultrasound (HIFU) ablation have been explored and investigated as bridging therapy before LT, aiming at reducing waiting list dropout rate and recurrence after LT, and improving post-transplant survival. Recently, stereotactic body radiation therapy, a relatively non-invasive modality also demonstrated comparable results in terms of objective tumor response, dropout rate and post-operative complications after LT. However all of these studies were retrospective studies and did not employ modern imaging modalities to evaluate the objective response after SBRT.<br><br>Stereotactic body radiation therapy (SBRT), with the use of highly conformal and precision radiation techniques with real-time tracing of patient and tumor position so as to offer a high radiation dose to the tumors while sparing the adjacent critical organs from necessary radiation.<br><br>In addition, accuate and reliable imaging tools are essential to accurate diagnosis and staging of HCC, so as to allow the treating surgeons/physicians to decide if patients are within LT criteria and to maximize the use of the precious liver to those who are in real need. Dual tracer positron-emission tomography with integrated computed tomography (PET-CT) scan using 18F-

	fluorodeoxyglucose (FDG) and 11C-acetate (ACC) was proven to be more sensitive and specific to FDG-alone PET-CT scan in HCC diagnosis and staging. On the other hand, magnetic resonance imaging (MRI) with gadoxetate disodium is more accurate and sensitive to gadolinium-enhanced MRI and contrast-enhanced CT scan in detecting HCC and differentiate it from other non-HCC causes.<br><br>Based on the above, we are carrying out a phase 2 single-arm study on using SBRT as a bridging therapy before liver transplantation for HCC patients within LT criteria, monitored by pretreatment and posttreatment dual-tracer PET-CT scan and MRI scan with gadoxetate disodium.
**Study Design**	This study is a prospective phase 2, single arm study assessing the efficacy and safety of SBRT as briding therapy in patients with HCC before LT, monitored by regular pretreatment and posttreatment dual-tracer PET-CT scan.<br><br>Radical radiotherapy will be delivered by stereotactic body radiation therapy (SBRT) with 35 to 50Gy in 5 fractions over 5 to 14 days.<br><br>Patient is mandatory to have histologically or radiologically confirmed HCC.<br><br>The co-primary endpoints of this study will be progression-free survival, and objective response. Patients will undergo tumor assessment with dual-tracer PET-CT scan and MRI scan with gadoxetate disodium at baseline, and then at 3 months and 6 months after SBRT and continuous interval dual-phase contrast CT scans every 3 months after SBRT for treatment response evaluation and surveillance by mRECIST, RECIST 1.1 and irRECIST until LT is done or when progressive disease develops.<br><br>Satefy assessments will include the incidence, nature, and severity of adverse events, changes in vital signs and laboratory abnormalities grade per National Cancer Institute Common Terminology Criteria for Adverse Events (NCI CTCAE), Version 4.0.
**Primary Objectives**	• Progression-free survival (PFS), calculated from start of SBRT to the date of radiologically confirmed progressive disease based on RECIST 1.1, mRECIST, PERCIST or death from any cause<br><br>• Best objective response as defined per RECIST, mRECIST, and PERCIST

**Secondary Objectives**	• Local control rate as defined by RECIST 1.1, mRECIST, and PERCIST<br><br>• Overall survival (OS), calculated from start of treatment to the date of death from any cause<br><br>• Safety and toxicity profiles as determined by Common Terminology Criteria for Adverse Events (CTCAE) version 4.0
**Exploratory Objectives**	• Prognostic factors of PFS and OS<br><br>• Predictive factors of objective response after SBRT<br><br>• Change in Child-Pugh scores before and after SBRT, especially the incidence of increase in Child-Pugh score by ≥2 points after SBRT
**Number of patient**	A total of 28 patients will be accured and 35 patients will be screened to allow a dropout rate of 20%
**Inclusion Criteria**	1. Patients must have histologically or radiologically confirmed HCC not amenable to curative resection. For radiological diagnosis of HCC, a contrast-enhanced computed tomography or magnetic resonance imaging is mandatory to demonstrate the early arterial enhancement in arterial phase and contrast washout in the porto-venous phase on the imaging<br><br>2. Capable of giving signed informed consent which includes compliance with the requirements and restrictions listed in the informed consent form (ICF) and in this protocol. Written informed consent and any locally required authorisation (e.g. Health Insurance Portability and Accountability Act in the US, Euripean Union [EU] Data Privacy Directive in the EU) obtained from the patient/legal representative prior to performing any protocol-related procedures, including screening evaluations<br><br>3. HCC lesions within UCSF criteria for LT<br><br>4. Be ≥ 18 years of age on day of signing informed consent<br><br>5. Have a performance status of 0 or 2 on the Eastern Cooperative Oncology Group (ECOG) Performance Scale.<br><br>6. A stage C or earlier HCC based on Barcelona Clinic Liver

Cancer (BCLC) staging system.

7. A Child-Pugh score of B8 or less.

8. Demonstrate adequate organ function as defined in Inclusion Criteria 9, all screening labs should be performed 28 days prior to study registration up to first dose of study drug.

9. Adequate serum hematological functions defined as:

- Absolute neutrophil count (ANC) ≥ 1.0 × 10^9/l

- Platelet ≥ 20 × 10^9/l

- Hemoglobin ≥ 8 g/dl

Adequate serum biochemistry functions defined as:

- Serum bilirubin ≤ 5.0 x institutional upper limit of normal (ULN). <<This will not apply to patients with confirmed Gilbert's syndrome (persistent or recurrent hyperbilirubinemia that is predominantly unconjugated in the absence of hemolysis or hepatic pathology), who will be allowed only in consultation with their physician.>>

- AST (SGOT)/ALT (SGPT) ≤ 6 × institutional upper limit of normal

- Albumin ≥ 25g/l

- Measured creatinine clearance (CL) > 40 ml/min or Calculated creatinine CL > 40 ml/min by the Cockcroft-Gault formula (Cockcroft and Gault 1976) or by 24-hour urine collection for determination of creatinine clearance:

Males:

$$\text{Creatinine CL (ml/min)} = \frac{\text{Weight (kg)} \times (140 - \text{Age})}{72 \times \text{serum creatinine (mg/dl)}}$$

Females:

$$\text{Creatinine CL (mL/min)} = \frac{\text{Weight (kg)} \times (140 - \text{Age})}{72 \times \text{serum creatinine (mg/dL)}} \times 0.85$$

	10. International Normalized Ratio (INR) or Prothrombin Time (PT) ≤ 2.0 × ULN unless subject is receiving anticoagulant therapy as long as PT or PTT is within therapeutic range of intended use of anticoagulants. Activated Partitional Thromboplastin Time (aPTT) ≤ 2.0 × ULN unless subject is receiving anticoagulant therapy as long as PT or PTT is within therapeutic range of intended use of anticoagulants.<br><br>11. Female subject of childbearing potential should have a negative urine or serum pregnancy within 24 hours of study enrollment up to administration of the dose of study drug. If the urine test is positive or cannot be confirmed as negative, a serum pregnancy test will be required.<br><br>12. Female subjects of childbearing potential should be willing to use 2 methods of birth control or be surgically sterile, or abstain from heterosexual activity for the course of the study through 31 weeks after the last dose of study medication. Subjects of childbearing potential are those who have not been surgically sterilized or have not been free from menses for > 1 year. The following age-specific requirements apply:<br><br>- Women < 50 years of age would be considered post-menopausal if they have been amenorrheic for 12 months or more following cessation of exogenous hormonal treatments and if they have luteinizing hormone and follicle-stimulating hormone levels in the post-menopausal range for the institution or underwent surgical sterilization (bilateral oophorectomy or hysterectomy).<br><br>- Women ≥ 50 years of age would be considered post-menopausal if they have been amenorrhoeic for 12 months or more following cessation of all exogenous hormonal treatments, had radiation-induced menopause with last menses > 1 year ago, had chemotherapy-induced menopause with last menses > 1 year ago, or underwent surgical sterilization (bilateral oophorectomy, bilateral salpingectomy or hysterectomy).<br><br>13. Male subjects should agree to use an adequate method of contraception starting with the first dose of study therapy through 31 weeks after the last dose of study therapy.<br><br>14. Patient is willing and able to comply with the protocol for the duration of the study including undergoing treatment and scheduled visits and examinations including follow up.<br><br>15. Must have a life expectancy of at least 12 weeks.

**Exclusion Criteria**	1. Has received any form of tumor resection of HCC.<br><br>2. Has received prior locoregional therapy including but not limited to radiofrequency ablation, microwave ablation, ethanol injection, transarterial chemoembolization and transarterial radioembolization.<br><br>3. Is currently participating in or has participated in a study of an investigational agent or using an investigational device within 4 weeks of the first dose of treatment or 5 half-lives, whichever is shorter.<br><br>4. Has a diagnosis of severe active scleroderma, lupus, other rheumatologic or autoimmune disease within the past 3 months before study recruitment. Patients with a documented history of clinically severe autoimmune disease or a syndrome requiring systemic steroids or immunosuppressive agents will not be allowed on this study. Subjects with vitiligo or resolved childhood asthma/atopy are an exception to this rule. Subjects that require intermittent use of bronchodilators or local steroid injections are not excluded from the study. Subjects with hypothyroidism stable on hormone replacement are not excluded from this study.<br><br>5. Has had a prior monoclonal antibody, immunotherapy or immune checkpoint inhibitors before recruitment into this study.<br><br>6. Has had prior chemotherapy or targeted small molecule therapy (including sorafenib, lenvatinib, or other anti-vascular endothelial growth factor inhibitor) before recruitment into this study.<br><br>7. Has a known additional malignancy that is progressing or requires active treatment. Exceptions include basal cell carcinoma of the skin, squamous cell carcinoma of the skin, indolent lymphomas, or in situ cervical cancer that has undergone potentially curative therapy<br><br>8. Has a history of prior solid organ transplants with or without any episodes of graft-versus-host disease.<br><br>9. Has a history of allogeneic bone marrow transplantation and peripheral stem cell rescue, with or without any episodes of graft-versus-host disease.

	10. Has known extra-hepatic metastases.<br><br>11. Has known carcinomatous meningitis (also known as leptomeningeal carcinomatosis)<br><br>12. Has an active infection requiring intravenous systemic therapy or hospital admission.<br><br>13. Has a history or current evidence of any condition, therapy, or laboratory abnormality, including psychiatric or substance abuse disorder, that might confound the results of the trial, interfere with the subject's participation for the full duration of the trial, or is not in the best interest of the subject to participate, in the opinion of the treating investigator.<br><br>14. Is pregnant or breastfeeding, or expecting to conceive or father children within the projected duration of the trial, starting with the screening visit through 31 weeks after the last dose of trial treatment.<br><br>15. Untreated hepatitis B infection. Patients with chronic hepatitis B infection (defined as HBsAg positive) are eligible if they have started anti-viral therapy for at least 1 month and is continuing anti-viral treatment throughout the whole duration of this study.<br><br>16. Has experienced Grade 4 toxicity on treatment with prior radiation if done before.<br><br>17. Prior systemic therapy utilizing an anti CTLA-4 or PD-1/PD-L1 agent or other forms of immunotherapy.<br><br>18. Has had prior radiation therapy (defined as > 0.5Gy) to the area planning to be treated with SBRT.
**Radiotherapy Planning**	Radical radiotherapy will be delivered by stereotactic body radiation therapy (SBRT) which is described as follows:<br><br>SBRT will be delivered by either a 10MV linear accelerator (TrueBeam, Varians Medical System, US), Tomotherapy (Accuray) in the treating institution. Four-dimensional (4D) or breath-hold CT scanning for tracking the tumor and liver positions and and radiation therapy planning is done with Real-time Patient Management (RPM) system (Varian Medical Systems, USA) or ExacTrac Adaptive Gating systems (Brainlab, AG, Germany) for motion detection. The gross tumor volume (GTV) with the co-registration of MRI and PET-CT images, and the motion of the

	tumor known as the internal target volume (ITV) (if any) are then contoured on the 4-dimensional CT or breath-hold CT images. The planning target volume (PTV) which encompasses the ITV with a 2-3mm margin, takes the set-up errors into account. Doses of SBRT range from 35Gy to 50Gy in 5 fractions over 5 to 14 days as specified by RTOG 1112 protocol, a phase 2 study investigating the use of sorafenib in combination with SBRT for inoperable HCC. The final dose is determined such that a maximum tumoricidal dose can be delivered to the tumors while the doses to the organs-at-risk are within the set tolerance limits. Pretreatment positional verification by a cone-beam CT scanner, on-board imaging and/or other imaging devices available in each treating institution is done every time before SBRT with necessary correction if the linear and rotational displacement is more than 2mm and 2 degrees respectively. In addition, interval treatment verification with on-board x-ray imaging and cone-beam CT scan during SBRT may be performed additionally for identification of intra-fractional tumor displacement.
**Safety Assessments**	Safety assessments will include the incidence, nature, and severity of adverse events, changes in vital signs and laboratory abnormalities grade per National Cancer Institute Common Terminology Criteria for Adverse Events (NCI CTCAE), version 4.0.
**Hypothesis**	1. We hypothesize that SBRT as bridging therapy before LT can achieve an objective response of 85%<br>2. We hypothesize that SBRT as bridging therapy<br>3. We hypothesize that SBRT is safe and effective in achieving a promising objective response and local control, rendering patients suitable for LT<br>4. We hypothesize that dual-tracer PET-CT and MRI with gadoxetate disodium can accurately diagnose and stage HCC patients to meet LT criteria and facilitate subsequent SBRT planning and monitor tumor response after SBRT
**Statistical Analysis Plan and Rationale for Number of Patient**	The sample size for this phase 2 study is based on the following assumptions:<br><br>• p0 = 60%, assumed tumor objective response after TACE or RFA<br><br>• p1 = 85%, expected tumor objective response after SBRT<br><br>With a two-sided alpha value of 0.05 and an 80% power of detection of statistical significance, 28 patients were required. Assuming a dropout rate of about 20%, 35 patients were required for eligibility screening in this study.

	Kaplan-Meier methods are used for survival analysis. Log-rank tests are performed for comparison of survival among different patient subgroups. Cox-regression with univariable and multivariable analyses are performed for prognostic factors of all prespecified study endpoints.<br><br>All statistical analyses are performed by Statistical Package for Social Sciences (SPSS) version 24 or R 3.6.1 or more advanced versions. Statistical significance is defined as *P* value < .05 (two-sided).
**Duration of Patient Participation**	The duration of treatment depends on the availability of LT after SBRT, typicaly ranging from 6 months to 4 years.

TABLE OF CONTENTS

1. INTRODUCTION 144
1.1 Background 144
1.1.1 Hepatocellular Carcinoma 144
1.1.2 Stereostactic Body Radiation Therapy 144
1.1.3 Magnetic resonance imaging with gadoxetate disodium 144
1.1.4 Dual-tracer positron emission tomography 144
1.1.5 Rationale for Conducting this Study 15
1.2 Research hypothesis 15
1.3 Benefit/risk assessment 15
1.3.1 Potential benefits of SBRT as bridging therapy 15
1.3.2 Potentail risks of SBRT as bridging therapy 15
2. STUDY OBJECTIVES 15
2.1 Primary Objectives 16
2.2 Secondary Objectives 16
2.3 Study Design 16
2.4 Study Participants 16
3. ELIGIBILITY CRITERIA 16
3.1 Inclusion Criteria 16
3.2 Exclusion Criteria 19
4. STUDY TREATMENT 20
4.1 Stereotactic body radiation therapy (SBRT) 20
4.2 Concomitant therapy 21
4.2.1 Dose modification for SBRT 21
5. DISCONTINUATION OF STUDY INTERVENTION AND PARTICIPANT WITHDRAWAL 21

5.1 Patient Discontinuation **22**
5.2 Study Treatment Discontinuation **22**
5.3 Study Discontinuation **22**
5.4 Withdrawal of Consent **23**
6. STUDY ASSESSMENTS AND PROCEDURES **23**
6.1 Informed Consent **24**
6.2 Inclusion/Exclusion Criteria **24**
6.3 Medical History **24**
6.4 Concomitant Medications **24**
6.4.1 Permitted concomitant medications 24
6.4.2 Excluded concomitant medications 25
6.5 Efficacy Assessments **25**
6.6 Physical Examination **27**
6.7 Clinical Safety Laboratory Assessments **27**
6.8 Safety Reporting **28**
6.8.1 Adverse Events (AE) 28
6.8.2 Serious Adverse Events (SAE) 29
6.8.3 Pregnancy Reports 29
6.8.4 Assessment of Causality of Adverse Events 30
6.8.5 Methods of recording Adverse Events 30
6.8.6 Procedure of Reporting Serious Adverse Events 32
7. RESTRICTIONS DURING THE STUDY AND CONCOMITANT TREATMENTS **32**
8. STATISTICAL PLAN **34**
8.1 Analysis Endpoints **34**
8.1.1 Efficacy Endpoint 34
8.1.2 Safety Endpoint 35

8.1.3 Exploratory 35

9. ETHICAL, REGULATORY & STUDY OVERSIGHT CONSIDERATIONS 35

9.1 Ethical Conduct of the Study 35

9.2 Responsibilities of the Investigator(s) 35

9.3 Subject Information 35

9.4 Compensation to Subjects 36

9.5 Ethics Committee or Institutional Review Board 36

10. STUDY MANAGEMENT 37

10.1 Data Quality Assurance 37

10.2 Direct Access to Source Data/Documents 37

10.3 Study File, Database and Archiving 38

APPENDIX 1 39

APPENDIX 2 44

APPENDIX 3 45

APPENDIX 4 46

APPENDIX 5 47

REFERENCES 51

1. INTRODUCTION

1.1 Background

1.1.1 Hepatocellular Carcinoma

Hepatocellular carcinoma (HCC) is one of the commonest solid malignancies in Asia Pacific region, primarily because of high incidence of hepatitis B infection in this locality.[1,2] Resection or liver transplantation (LT) is the standard treatment for operable/resectable disease.[3,4] However the waiting time for LT can be very long, especially in countries/regions where organ shortage is a serious problem. Bridging therapy like transarterial chemoembolization (TACE), radiofrequency ablation or more recently transarterial radioembolization have been investigated and evaluated in HCC patients before LT.[5-7] Stereotactic body radiation therapy (SBRT) has also been explored and evaluated in this study. However so far, there has been no prospective trial on SBRT as bridging therapy before LT, though retrospective studies revealed promising tumor response and safety profiles.[7-15] There are no studies on using magnetic resonance imaging (MRI) and dual-tracer PET-CT scan in HCC diagnosis and treatment monitoring in this setting.

1.1.2 Stereostactic Body Radiation Therapy

Stereotactic body radiation therapy (SBRT), with the use of highly conformal and precision radiation techniques with real-time tracing of patient and tumor position so as to offer a high radiation dose to the tumors while sparing the adjacent critical organs from necessary radiation.

1.1.3 Magnetic resonance imaging (MRI) with gadoxetate disodium

Contrast-enhanced triphasic computed tomography (CT) scan has been regarded as the standard imaging tool in HCC diagnosis and treatment monitoring.[16] MRI with gadolinium contrast injection demonstrated comparable sensitivity and specificity with contrast-enhanced computed tomography for HCC lesions > 2cm. The addition of liver specific contrast gadoxetate disodium further improves detection of 1-2cm tumors, demonstrating 92.1% accuracy based on the Milan and UNOS guidelines.[17] Two recent meta-analysis revealed that gadoxetate disodium-enhanced MRI demonstrated higher sensitivity and diagnostic accuracy than contrast-enhanced CT scan, especially for lesions < 2cm.[18,19]

1.1.4 Dual-tracer positron-emission tomography with integrated computed tomography (PET-CT)

PET-CT with 18F-fluorodeoxyglucose (FDG) has also been evaluated in HCC diagnosis and surveillance after treatment. However, FDG is just able to detect poorly differentiated HCC only. Dual-tracer (11C-acetate [ACC] and 18F-fluorodeoxyglucose [FDG]) positron-emission tomography with integrated CT (PET-CT) was proven more sensitive and specific than FDG-alone PET-CT in HCC diagnosis and surveillance.[20,21] In particular, dual-tracer PET-CT was found superior than contrast-enhanced CT scan in diagnosing and selecting

patients for LT, so that the liver grafts were given only to those who really fulfilled the LT criteria.[22]

1.1.5 Rationale for conducting this study

In view of the above with, we are proposing a phase 2, single-arm study on SBRT as a bridging therapy before LT for HCC patients. MRI with gadoxetate disodium and dual-tracer PET-CT scan will be used as HCC diagnosis as well as treatment response evaluation following SBRT until LT is done or when progressive disease is documented. By doing so, it is hoped that these two imaging modalities will help identify those who truly fulfilled LT criteria so that LT will only be performed to those who are accurately and reliably diagnosed with the most contemporary imaging techniques.

1.2 Research hypothesis

1. We hypothesize that SBRT as bridging therapy before LT can achieve an objective response of 85%
2. We hypothesize that SBRT as bridging therapy
3. We hypothesize that SBRT is safe and effective in achieving a promising objective response and local control, rendering patients suitable for LT
4. We hypothesize that dual-tracer PET-CT and MRI with gadoxetate disodium can accurately diagnose and stage HCC patients to meet LT criteria and facilitate subsequent SBRT planning and monitor tumor response after SBRT

1.3.1 Potential benefits of this study

We expect that SBRT as bridging therapy can produce an 85% objective response rate, so that enough time is allowed for the patients to wait for LT. Besides, use of dual-tracer PET-CT and magnetic resonance imaging with gadoxetate disodium can provide more accurate diagnosis, staging for HCC and treatment response monitoring after SBRT so that LT will be allocated to those who fulfills the LT criteria.

1.3.2 Potential risks of this study

Patients undergoing SBRT may have a risk of radiation-induced liver injury (RILD), which depends on the radiation dose and fractionation to the liver. Classic RILD is a syndrome occurring most often within 2 months following radiation therapy, consisting of anicteric hepatomegaly and elevation of liver enzymes (ALP>AST). Treatment for RILD is usually symptomatic though it may progress to liver failure, despite maximal supportive care. Classic RILD is uncommon in modern radiation therapy series like SBRT, when the dose to the liver can be kept below recommended levels. Non-classic RILD, referring to any decline in liver function or liver toxicity, excluding classic RILD (e.g. elevated transaminases or reduction of Child-Pugh score) is more common in HCC patients treated with RT. It is more likely in patients with a higher Child-Pugh score at baseline and in those with more advanced tumors requiring a larger volume of liver to be irradiated.

2. STUDY OBJECTIVES

2.1 Primary Objectives

- Progression-free survival (PFS), calculated from start of SBRT to the date of radiologically confirmed progressive disease based on RECIST, mRECIST, and PERCIST or death from any cause
- Best objective response as defined by RECIST 1.1,[23] mRECIST,[24] and PERCIST[25]

2.2 Secondary Objectives

- Local control rate as defined by RECIST 1.1, mRECIST, and PERCIST
- Overall survival (OS), calculated from start of treatment to the date of death from any cause
- Safety and toxicity profiles as determined by Common Terminology Criteria for Adverse Events (CTCAE) version 4.0

2.3 Study Design

This study is a prospective phase II, single-arm study assessing the efficacy and safety of SBRT as bridging therapy in HCC patients who are awaiting LT.

2.4 Study Participants

A total of 35 patients will be screened and 28 patients will be recruited and analysed to estimate the potential benefits and safety profiles of SBRT as bridging therapy before LT.

3. ELIGIBILITY CRITERIA

3.1 Inclusion Criteria

1. Patients must have histologically or radiologically confirmed HCC not amenable to curative resection. For radiological diagnosis of HCC, a contrast-enhanced computed tomography or magnetic resonance imaging is mandatory to demonstrate the early arterial enhancement in arterial phase and contrast washout in the porto-venous phase on the imaging.

2. Capable of giving signed informed consent which includes compliance with the requirements and restrictions listed in the informed consent form (ICF) and in this protocol. Written informed consent and any locally required authorization (e.g. Health Insurance Portability and Accountability Act in the US, European Union [EU] Data Privacy Directive in the EU) obtained from the patient/legal representative prior to performing any protocol-related procedures, including screening evaluations.

3. HCC lesions within University of California, San Francicso (UCSF) criteria for LT (solitary tumor ≤ 6.5cm, up to 3 tumors with none > 4.5cm, and total tumor diameter ≤ 8cm).

4. Be ≥ 18 years of age on day of signing informed consent.

5. Have a performance status of 0 to 2 on the Eastern Cooperative Oncology Group (ECOG) Performance Scale.

6. A stage C or earlier HCC based on Barcelona Clinic Liver Cancer (BCLC) staging system.

7. A Child-Pugh score of B8 or less.

8. Demonstrate adequate organ function as defined in Inclusion Criteria 7, all screening labs should be performed 28 days prior to study registration up to the first dose of study drug.

9. Adequate serum hematological functions defined as:
   - Absolute neutrophil count (ANC) ≥ 1.0 × 10^9/l
   - Platelet ≥ 20 × 10^9/l
   - Hemoglobin ≥ 8 g/dl

   Adequate serum biochemistry functions defined as:
   - Serum bilirubin ≤ 5.0 x institutional upper limit of normal (ULN). <<This will not apply to patients with confirmed Gilbert’s syndrome (persistent or recurrent hyperbilirubinemia that is predominantly unconjugated in the absence of hemolysis or hepatic pathology), who will be allowed only in consultation with their physician.>>

   - AST (SGOT)/ALT (SGPT) ≤ 6.0 x institutional upper limit of normal unless liver metastases are present, in which case it must be ≤ 6.0 × ULN.

   - Measured creatinine clearance (CL) > 40 ml/min or Calculated creatinine CL > 40 mL/min by the Cockcroft-Gault formula (Cockcroft and Gault 1976) or by 24-hour urine collection for determination of creatinine clearance:

Males:

$$\text{Creatinine CL (mL/min)} = \frac{\text{Weight (kg)} \times (140 - \text{Age})}{72 \times \text{serum creatinine (mg/dL)}}$$

Females:

$$\text{Creatinine CL (mL/min)} = \frac{\text{Weight (kg)} \times (140 - \text{Age})}{72 \times \text{serum creatinine (mg/dL)}} \times 0.85$$

10. International Normalized Ratio (INR) or Prothrombin Time (PT) ≤ 2.0 × ULN unless subject is receiving anticoagulant therapy as long as PT or PTT is within therapeutic range of intended use of anticoagulants. Activated Partitional Thromboplastin Time (aPTT) ≤ 2.0 × ULN unless subject is receiving anticoagulant therapy as long as PT or PTT is within therapeutic range of intended use of anticoagulants.

11. Female subject of childbearing potential should have a negative urine or serum pregnancy within 24 hours of study enrollment up to administration of the dose of study drug. If the urine test is positive or cannot be confirmed as negative, a serum pregnancy test will be required.

12. Female subjects of childbearing potential should be willing to use 2 methods of birth control or be surgically sterile, or abstain from heterosexual activity for the course of the study through 31 weeks after the last dose of study medication. Subjects of childbearing potential are those who have not been surgically sterilized or have not been free from menses for > 1 year. The following age-specific requirements apply:

    - Women < 50 years of age would be considered post-menopausal if they have been amenorrheic for 12 months or more following cessation of exogenous hormonal treatments and if they have luteinizing hormone and follicle-stimulating hormone levels in the post-menopausal range for the institution or underwent surgical sterilization (bilateral oophorectomy or hysterectomy).

    - Women ≥ 50 years of age would be considered post-menopausal if they have been amenorrheic for 12 months or more following cessation of all exogenous hormonal treatments, had radiation-induced menopause with last menses >1 year ago, had chemotherapy-induced menopause with last menses >1 year ago, or underwent surgical sterilization (bilateral oophorectomy, bilateral salpingectomy or hysterectomy).

13. Male subjects should agree to use an adequate method of contraception starting with the first dose of study therapy through 31 weeks after the last dose of study therapy.

14. Patient is willing and able to comply with the protocol for the duration of the study including undergoing treatment and scheduled visits and examinations including follow up.

15. Must have a life expectancy of at least 12 weeks.

3.2 Exclusion Criteria

1. Has received any form of tumor resection of HCC.

2. Has received prior locoregional therapy including but not limited to radiofrequency ablation, microwave ablation, ethanol injection, transarterial chemoembolization and transarterial radioembolization.

3. Is currently participating in or has participated in a study of an investigational agent or using an investigational treatment/device within 4 weeks of the first dose of treatment or 5 half-lives, whichever is shorter.

4. Has a diagnosis of severe active scleroderma, lupus, other rheumatologic or autoimmune disease within the past 3 months before study recruitment. Patients with a documented history of clinically severe autoimmune disease or a syndrome requiring systemic steroids or immunosuppressive agents will not be allowed on this study. Subjects with vitiligo or resolved childhood asthma/atopy are an exception to this rule. Subjects that require intermittent use of bronchodilators or local steroid injections are not excluded from the study. Subjects with hypothyroidism stable on hormone replacement are not excluded from this study.

5. Has had a prior monoclonal antibody, immunotherapy or immune checkpoint inhibitors before recruitment into this study.

6. Has had prior chemotherapy or targeted small molecule therapy (including sorafenib, lenvatinib, or other anti-vascular endothelial growth factor inhibitor) before recruitment into this study.

7. Has a known additional malignancy that is progressing or requires active treatment. Exceptions include basal cell carcinoma of the skin, squamous cell carcinoma of the skin, indolent lymphomas, or in situ cervical cancer that has undergone potentially curative therapy

8. Has a history of prior solid organ transplants with or without any episodes of graft-versus-host disease.

9. Has a history of allogeneic bone marrow transplantation and peripheral stem cell rescue, with or without any episodes of graft-versus-host disease.

10. Has known extra-hepatic metastases.

11. Has known carcinomatous meningitis (also known as leptomeningeal carcinomatosis).

12. Has an active infection requiring intravenous systemic therapy or hospital admission.

13. Has a history or current evidence of any condition, therapy, or laboratory abnormality, including psychiatric or substance abuse disorder, that might confound the results of the trial, interfere with the subject's participation for the full duration of the trial, or is not in the best interest of the subject to participate, in the opinion of the treating investigator.

14. Is pregnant or breastfeeding, or expecting to conceive or father children within the projected duration of the trial, starting with the screening visit through 31 weeks after the last dose of trial treatment.

15. Untreated hepatitis B infection. Patients with chronic hepatitis B infection (defined as HBsAg positive) are eligible if they have started anti-viral therapy for at least 1 month and is continuing anti-viral treatment throughout the whole duration of this study.

16. Has experienced Grade 4 toxicity on treatment with prior radiation if done before.

17. Prior systemic therapy utilising an anti CTLA-4 or PD-1/PD-L1 agent or other forms of immunotherapy.

18. Has had prior radiation therapy (defined as > 0.5Gy) to the area planning to be treated with SBRT.

4. STUDY TREATMENT

4.1 Stereotactic Body Radiation Therapy (SBRT)

Radical radiotherapy will be delivered by SBRT which is described as follows:

SBRT will be delivered by either a 10MV linear accelerator (TrueBeam, Varians Medical System, Palo Alto, USA) in the treating institution within 2 weeks of baseline investigations. Four-dimensional (4D) CT scanning for tracking the respiratory phases and radiation therapy planning is done with Real-time Patient Management (RPM) system (Varian Medical Systems, USA) or ExacTrac Adaptive Gating systems (Brainlab, AG, Germany) for motion detection. The gross tumor volume (GTV) with the co-registration of MRI and PET-CT images, and the motion of the tumor known as the internal target volume (ITV) are then contoured on the 4D CT images. The planning target volume (PTV) which encompasses the ITV with a 2–3mm margin, takes the set-up errors into account. Doses of SBRT range from 35Gy to 50Gy in 5 fractions over 5 to 14 days as adapted and modified from RTOG 1112 protocol,[27] a phase 2 study investigating the use of sorafenib in combination with SBRT for inoperable HCC. The final dose is determined such that a maximum tumoricidal dose can be delivered to the tumors while the doses to the organs-at-risk are within the set tolerance

limits. Pretreatment positional verification by a cone-beam CT scanner, on-board imaging or other imaging devices available in each treating institution is done every time before SBRT with necessary correction if the linear and rotational displacement is more than 2mm and 2 degrees respectively. In addition, interval treatment verification with on-board x-ray imaging during SBRT may be performed additionally for identification of intra-fractional tumor displacement.

The details of target volume nomenclature and delineation as well as the acceptance criteria of SBRT are outlined in APPENDIX 1.

4.2 Concomitant therapy

Prophylactic and/or rescue anti-emetics with aprepitant, 5-HT3 antagonists, metoclopramide as well as antacids, anti-H2 antagonists and proton pump inhibitors are allowed before, during and after SBRT as per local treating institution's protocols are allowed.

Other medication, which is considered necessary for the patient's safety and well being, may be given at the discretion of the investigator.

4.2.1 Dose Modification for SBRT

SBRT should be withhold when grade ≥ 3 toxicity (based on CTCAE version 4.0) develops. It can be only be resumed if the toxicity returns to grade ≤ 1.

If radiotherapy had to be interrupted more than 14 days because of toxicity or other reasons, the patient will be withdrawn from the study.

5. DISCONTINUATION OF STUDY INTERVENTION AND PARTICIPANT WITHDRAWAL

Discontinuation of treatment intervention dose not represent withdrawal from the study. As certain data on clinical events beyond study intervention discontinuation maybe important to the study, they must be collected through the participant's last scheduled follow-up, even if the participant has discontinued study intervention. Therefore, all participants who discontinue study intervention prior to completion of the protocol-specified treatment period will still continue to participate in the study for survival follow-up.

5.1 Patient Discontinuation

Patient have the right to voluntarily withdraw from the study at any time for any reason. In addition, the investigator has the right to withdraw a patient from the study at any time. Reasons for withdrawal from the study may include but are not limited to the following:

- Patient withdrawal of consent at any time
- Any medical condition that the investigator or sponsor determines may jeopardize the patient's safety if he or she continues in the study
- Investigator or sponsor determines it is in the best interest of the patient
- Patient non-compliance

Every effort should be made to obtain information on patients who withdraw from the study. The primary reason for withdrawal from the study should be documented. However, patients will not be followed for any reason after consent has been withdrawn.

5.2 Study Treatment Discontinuation

Permanent discontinuation of study treatment if one of the following occurs:

- Patient decision
- Loss to follow-up
- Severe non-compliance to the study protocol
- One or more of the exclusion criteria being met at study entry
- Adverse event contraindicating further treatment
- Initiation of alternative bridging therapy including locoregional therapy and/or anticancer therapy
- Confirmed disease progression
- Pregnancy or intent to become pregnant

5.3 Study Discontinuation

The investigators have the right to terminate this study at any time. Reasons for terminating the study may include but are not limited to the following:

- The incidence or severity of adverse events in this or other studies indicates a potential health hazard to patients
- Patient enrollment is unsatisfactory
- Excessively slow recruitment
- Poor protocol adherence
- Inaccurate or incomplete data recording
- Non-compliance with the International Conference on Harmonization (ICF) guideline for Good Clinical Practice

5.4 Withdrawal of Consent

Patients are free to withdraw from the study at any time without prejudice to further treatment.

Patients who withdraw consent for further participation in the study will not receive SBRT (if not done), but will continue to be observed during future follow-up visits for progressive

disease and survival, which will continue until the end of the study unless the patient has expressly withdrawn their consent to survival follow-up. Note that the patient may be offered additional tests or tapering of treatment to withdraw safely.

A patient who withdraws consent will always be asked about the reason(s) for withdrawal and the presence of any adverse events (AEs). The Investigator will follow up AEs outside of the clinical study.

If a patient withdraws consent, they will be specifically asked if they are withdrawing consent to:

- all further participation in the study including any further follow up (e.g., survival contact telephone calls)
- withdrawal of consent to the use of their study generated data
- withdrawal to the use of any samples (if any)

6. STUDY ASSESSMENTS AND PROCEDURES

The following procedures will be performed during the screening visit:

- Informed Consent
- Review of eligibility criteria
- Medical history and demographics
- Complete physical exam
- ECOG Performance Status
- Vitals signs, weight and height
- Blood for Hepatitis B viral (HBV) serology including surface antigens of Hepatitis B virus (HBsAg), antibody against the surface antigen (Anti-HBs) and HBV DNA, and Hepatitis C viral (HCV) serology with anti-HCV.
- Review of prior/concomitant medications
- Imaging by MRI with gadoxetate disodium, dual-tracer (FDG and ACC) PET-CT with contrast-enhanced CT scan. The scanning algorithms of MRI with gadoxetate disodium and dual-tracer PET-CT scan are shown in APPENDIX 2 and APPENDIX 3.

- Clinical laboratory tests for:
    - Hematology
    - Clinical biochemistry
    - Coagulation profile (PT, PTT, INR)
    - Creatinine clearance

- Serum pregnancy test (for women of childbearing potential only)
- Hepatitis serologies including Hepatitis B virus (HBV) and Hepatitis C virus (HCV) as mentioned above.

6.1 Informed Consent

The investigator or designee must obtain documented consent from each potential participant or each participant’s legally acceptable representative prior to participating in a clinical study. Consent must be documented by the participant’s dated signature or participant’s legally acceptable representative’s dated signature on a consent form along with the dated signature of the person conducting the consent discussion.

A copy of the signed and dated consent form should be given to the participant before participation in the study.

6.2 Inclusion/Exclusion Criteria

All inclusion and exclusion criteria will be reviewed by the investigator to ensure that the participant is eligible for the study before starting study intervention.

6.3 Medical History

Medical history will be obtained by the investigator or designee. The medical history will collect all active conditions and any condition diagnosed within the prior 10 years that the investigator considers to be clinically significant.

6.4 Concomitant Medications

Therapies considered necessary for the patient’s well-being may be administered at the discretion of the Principle Investigator and/or co-investigators. All medications administered at the start of study drug through last dose of study drug must be recorded on the appropriate source documents.

6.4.1 Permitted concomitant medications

**Supportive medication/class of drug:**	**Usage:**
Concomitant medications or treatments (e.g., acetaminophen or diphenhydramine) deemed necessary to provide adequate prophylactic or supportive care, except for those medications identified as “prohibited,” as listed above	To be administered as prescribed by the Investigator

Best supportive care (including antibiotics, nutritional support, correction of metabolic disorders, optimal symptom control, and pain management [including palliative radiotherapy to non-target lesions, etc])	Should be used, when necessary, for all patients
Inactivated viruses, such as those in the influenza vaccine	Permitted

6.4.2 Excluded concomitant medications

**Prohibited medication/class of drug:**	**Usage:**
Any investigational anticancer therapy other than those under investigation in this study	Should not be given concomitantly whilst the patient is on study treatment
mAbs against CTLA-4, PD-1, or PD-L1 other than those under investigation in this study	Should not be given concomitantly whilst the patient is on study treatment
Any concurrent chemotherapy, targeted therapy, immunotherapy, biologic or hormonal therapy, or any forms of radiotherapy for cancer treatment other than those under investigation in this study	Should not be given concomitantly whilst the patient is on study treatment. (Concurrent use of hormones for non-cancer-related conditions [e.g., insulin for diabetes and hormone replacement therapy] is acceptable. Local treatment of isolated lesions, excluding target lesions, for palliative intent is acceptable [e.g., by local surgery or radiotherapy])
Herbal and natural remedies which may have immune-modulating effects	Should not be given concomitantly unless agreed by the sponsor

6.5 Efficacy Assessments

Screening assessments must include magnetic resonance imaging (MRI) with gadoxetate disodium as well as dual-tracer PET-CT with contrast enhanced triphasic CT scan of the liver for tumor contouring and treatment planning for SBRT.

Follow-up imaging with MRI with gadoxetate disodium injection and dual-tracer PET-CT scan with contrast-enhanced triphasic CT scans every 3 months after SBRT will be arranged for treatment response evaluation by RECIST 1.1,[23] mRECIST,[24] and PERCIST[25] (APPENDIX 4 and APPENDIX 5) and surveillance for recurrence/relapse.

At the investigator’s discretion, scans should be repeated at any time if progressive disease is suspected.

Confirmation of progression guidelines are set for the following reasons:

- for patient management and treatment decisions
- when scans are evaluated by two independent radiologists.

Confirmed objective disease progression refers to either of the following scenarios: 1. clinical progression/deterioration followed by a radiologic verification scan (progressive disease [PD] by RECIST 1.1,[23] mRECIST,[24] and PERCIST[25]); or 2. in the absence of significant clinical deterioration, radiologic PD by RECIST 1.1, mRECIST, and PERCIST followed by a second radiologic confirmation scan with PD assessed according to the specific confirmation of progression criteria listed below. The confirmatory scan should occur preferably at the next scheduled imaging visit and no earlier than 4 weeks following the date of the immediate prior assessment of PD.

Immediate prior radiologic progression would be considered confirmed if any the following criteria are met in the confirmatory scan:

- ≥ 20% increase in the sum diameters of target lesions (TLs) compared with the nadir at 2 consecutive visits, with an absolute increase of at least 5 mm in sum of diameters compared to nadir,

- and/or significant progression (worsening) of non-target lesions (NTLs) and/or of pre-existing new lesions at the confirmatory scan time-point compared with the immediate prior time-point (Note: Pre-existing new lesions are evaluated as NTLs at the confirmatory scan time-point),

- and/or additional new unequivocal lesions at the confirmatory scan time-point.

If progression is not confirmed, in the absence of significant clinical deterioration, then the patient should continue the study and on-treatment assessments until the next PD which will also require a follow-up confirmation scan. If the first PD is not confirmed by the immediate next scan, then the Investigator should not change the PD assessment of the first scan.

If a patient discontinues treatment (and/or receives a subsequent anticancer therapy) prior to radiologic progression, then the patient should still continue to be followed until confirmed objective disease progression.

Following confirmed progression, patients should continue to be followed up for survival every 2-3 months until death or until patient refusal.

Please refer to APPENDIX 4 and APPENDIX 5 for the definitions of objective response by RECIST 1.1,[23] mRECIST,[24] and PERCIST.[25] The method of determination of metabolic tumor volume (MTV) and total lesion glycolysis (TLG) and total lesion activity (TLA) based on PET-CT scan with FDG and ACC respectively for subsequent exploratory statistical analyses were was described in APPENDIX 5.

6.6 Physical Examination

A complete physical examination should be performed at baseline, during SBRT until 3 months after SBRT, including height and weight measurements, and examinations of the respiratory system, abdomen and central nervous system. At subsequent visits, limited, symptom-directed physical examinations should be performed. Changes from baseline abnormalities should be recorded in patient notes.

Performance status will be assessed using ECOG performance status at each visit based on the following:

0 Fully active, able to carry on all pre-disease performance without restriction

1 Restricted in physically strenuous activity but ambulatory and able to carry out work of a light or sedentary nature, e.g., light house work, office work

2 Ambulatory and capable of all selfcare but unable to carry out any work activities; up and about more than 50% of waking hours

3 Capable of only limited selfcare; confined to bed or chair more than 50% of waking hours

4 Completely disabled; cannot carry on any selfcare; totally confined to bed or chair

5 Dead

6.7 Clinical Safety Laboratory Assessments

All recruited patients shall undergo baseline screening investigations including blood tests for serum hematology, biochemistry, clotting profile, alfa-feto protein and hepatitis B and C serology. All patients will be monitored once weekly during SBRT for any side effects, then monthly for 3 months followed by every 2-3 months with the aforementioned blood tests for serum hematology, biochemistry and clotting profile, alfa-feto protein and hepatitis B and C serology. The laboratory variables to be measured are presented in the following tables:

Hematology

Platelet Count	Basophils
Red Blood Cell Count	Eosinophils
Hemoglobin	Lymphocytes
Hematocrit	Monocytes
	Neutrophils

Clinical biochemistry (serum)

Albumin	Glucose
Alkaline phosphatase	Lactate dehydrogenase (LDH)
Alanine aminotransferase	Potassium
Aspartate aminotransferase	Sodium
Calcium	Total bilirubin
Chloride	Total bilirubin
Creatinine	Total protein
Gamma glutamyltransferase (GGT)	Urea
Globulin	

Tests for ALT, AST, alkaline phosphatase, and total bilirubin must be conducted and assessed concurrently. If total bilirubin is ≥ 2 × upper limit of normal (and no evidence of Gilbert's syndrome) then fractionate into direct and indirect bilirubin.

[b] Bicarbonate (where available), chloride, creatinine clearance, gamma glutamyltransferase, and magnesium testing are to be performed at baseline, on Day 0 (unless all screening laboratory clinical chemistry assessments are performed within 3 days prior to Day 0), and if clinically indicated.

[c] Creatinine Clearance will be calculated by data management using Cockcroft-Gault (using actual body weight).

**Clotting profile**	
INR	Prothrombin time<br>Activated partial thromboplastin time

**Hepatitis B and C serology**	
HBsAg<br>HBeAg, Anti-HBc	Anti-HBs<br>for hepatitis B<br>Anti-HCV for hepatitis C

Those patients who are found to be current or past hepatitis B or C carries must receive adequate anti-viral therapy as per local institution's routine clinical practice.

All patients with Grade 3 or 4 laboratory values at the time of completion or discontinuation from this study must have further tests performed until the laboratory values have returned to Grade 1 or 2, unless these values are not likely to improve because of the underlying disease.

6.8 Safety Reporting

6.8.1 Adverse Events (AE)

The International Conference on Harmonization (ICH) Guideline for Good Clinical Practice (GCP) E6(R1) defines an AE as:

Any untoward medical occurrence in a patient or clinical investigation patient administered a pharmaceutical product and which does not necessarily have a causal relationship with this treatment. An AE can therefore be any unfavorable and unintended sign (including an abnormal laboratory finding), symptom, or disease temporally associated with the use of a medicinal product, whether or not considered related to the medicinal product.

An AE includes but is not limited to any clinically significant worsening of a patient's pre-existing condition. An abnormal laboratory finding that requires an action or intervention by the investigator, or a finding judged by the investigator to represent a change beyond the range of normal physiologic fluctuation, should be reported as an AE.

Adverse events may be treatment emergent (i.e., occurring after initial receipt of investigational product) or nontreatment emergent. A nontreatment-emergent AE is any new

sign or symptom, disease, or other untoward medical event that begins after written informed consent has been obtained but before the patient has received investigational product.

Elective treatment or surgery or preplanned treatment or surgery (that was scheduled prior to the patient being enrolled into the study) for a documented pre-existing condition, that did not worsen from baseline, is not considered an AE (serious or nonserious). An untoward medical event occurring during the prescheduled elective procedure or routinely scheduled treatment should be recorded as an AE or SAE.

The term AE is used to include both serious and non-serious AEs.

6.8.2 Serious Adverse Events (SAE)

A serious adverse event is an AE occurring during any study phase (i.e., screening, run-in, treatment, wash-out, follow-up), at any dose of the study drugs that fulfils one or more of the following criteria:

- Results in death
- Is immediately life-threatening
- Requires in-patient hospitalization or prolongation of existing hospitalization
- Results in persistent or significant disability or incapacity
- Is a congenital abnormality or birth defect in offspring of the patient
- Is an important medical event that may jeopardize the patient or may require medical intervention to prevent one of the outcomes listed above.
    - Medical or scientific judgment should be exercised in deciding whether expedited reporting is appropriate in this situation. Examples of medically important events are intensive treatment in an emergency room or at home for allergic bronchospasm, blood dyscrasias, or convulsions that do not result in hospitalizations; or development of drug dependency or drug abuse.

The causality of SAEs (their relationship to all study treatment/procedures) will be assessed by the investigator(s) and communicated to local Institutional Review Board.

6.8.3 Pregnancy Reports

If a patient becomes pregnant before or during SBRT, the planned or ongoing SBRT should be discontinued immediately.

Male patients should refrain from fathering a child or donating sperm during the study and for at least 180 days after SBRT. They should also seek the medical advice from the Investigators in advance before they plan to father a child.

Pregnancy of the patient's partner is not considered to be an AE. However, the outcome of all pregnancies (spontaneous miscarriage, elective termination, ectopic pregnancy, normal birth, or congenital abnormality), if possible, be followed up and documented.

Where a report of pregnancy is received, prior to obtaining information about the pregnancy, the Investigator must obtain the consent of the patient's partner. Therefore, the local study team should adopt the generic ICF template in line with local procedures and submit it to the relevant Institutional Review Boards (IRBs)/Ethics Committee (EC) prior to use.

6.8.4 Assessment of Causality of Adverse Events

Investigator(s) should use their knowledge of the patient, the circumstances surrounding the event, and an evaluation of any potential alternative causes to determine whether an adverse event is considered to be related to the study treatment, indicating "Yes" or "No" accordingly. The following guidance should be taken into consideration:

- Temporal relationship of event onset to SBRT or LT
- Known association of the event with the disease under study
- Presence of risk factors in the patient or use of concomitant medications known to increase the occurrence of the event
- Presence of non-treatment-related factors that are known to be associated with the occurrence of the event

6.8.5 Methods of Recording Adverse Events

AEs and SAEs will be collected from the time of the patient signing the informed consent form until the follow-up period is completed. If an event that starts post the defined safety follow up period noted above is considered to be due to a late onset toxicity to study drug then it should be reported as an AE or SAE as applicable.

During the course of the study, all AEs and SAEs should be proactively followed up for each patient for as long as the event is ongoing. Every effort should be made to obtain a resolution for all events, even if the events continue after the patient has discontinued study drug or the study has completed.

Any AEs that are unresolved at the patient's last visit in the study are followed up by the Investigator for as long as medically indicated.

The following variables will be collected for each AE:

In addition, the following variables will be collected for SAEs as applicable:

- AE (verbatim)
- The date when the AE started and stopped
- The maximum CTCAE grade reported
- Changes in CTCAE grade
- Whether the AE is serious or not
- Investigator causality rating against the treatment or procedure(s) (yes or no)

- Action taken with regard to treatment
- Administration of treatment for the AE
- Outcome

In addition, the following variables will be collected for SAEs:

- Date the AE met criteria for SAE
- Date the Investigator became aware of the SAE
- Seriousness criteria fulfilled
- Date of hospitalization
- Date of discharge
- Probable cause of death
- Date of death
- Whether an autopsy was performed
- Causality assessment in relation to study treatment or procedure(s)
- Causality assessment in relation to other medication
- Description of the SAE

The grading scales found in the NCI CTCAE version 4.0 will be utilized for all events with an assigned CTCAE grading. For those events without assigned CTCAE grades, the recommendation in the CTCAE criteria that converts mild, moderate, and severe events into CTCAE grades should be used. A copy of the CTCAE version 4.0 can be downloaded from the Cancer Therapy Evaluation Program website (http://ctep.cancer.gov).

Events, which are unequivocally due to disease progression, should not be reported as an AE during the study.

Adverse events and serious adverse events will be recorded from time of signature of informed consent, throughout the treatment period and including the follow-up period.

During the course of the study all AEs and SAEs should be proactively followed up for each patient. Every effort should be made to obtain a resolution for all events, even if the events continue after discontinuation/study completion.

If a patient discontinues from treatment for reasons other than disease progression, and therefore continues to have tumor assessments, drug or procedure-related SAEs must be captured until the patient is considered to have confirmed PD and will have no further tumor assessments.

The investigator is responsible for following all SAEs until resolution, until the patient returns to baseline status, or until the condition has stabilized with the expectation that it will remain chronic, even if this extends beyond study participation.

6.8.6 Procedure of Reporting Serious Adverse Events

All SAEs have to be reported, whether or not considered causally related to the treatment, or to the study procedure(s).

For fatal or life-threatening AEs where important or relevant information is missing, active follow-up is undertaken immediately. Investigators or other site personnel inform representatives of the local institutional review board of any follow-up information on a previously reported SAE immediately, or no later than 24 hours of when he or she becomes aware of it.

7. RESTRICTIONS DURING THE STUDY AND CONCOMITANT TREATMENTS

The following restrictions apply while the patient is receiving study treatment and for the specified times before and after:

Female patient of child-bearing potential:

- Female patients of childbearing potential who are not abstinent and intend to be sexually active with a non-sterilized male partner must use at least 1 highly effective method of contraception (Table 1) from the time of screening throughout the total duration of the study. Non-sterilised male partners of a female patient of childbearing potential must use male condom plus spermicide throughout this period. Cessation of birth control after this point should be discussed with a responsible physician. Periodic abstinence, the rhythm method, and the withdrawal method are not acceptable methods of birth control. Female patients should also refrain from breastfeeding throughout this period.

Male patients with a female partner of childbearing potential:

- Non-sterilized male patients who are not abstinent and intend to be sexually active with a female partner of childbearing potential must use a male condom plus spermicide from the time of screening throughout the total duration of the study. However, periodic abstinence, the rhythm method, and the withdrawal method are not acceptable methods of contraception. Male patients should refrain from sperm donation throughout this period.

- Female partners (of childbearing potential) of male patients must also use a highly effective method of contraception throughout this period (Table 1).

N.B Females of childbearing potential are defined as those who are not surgically sterile (ie, bilateral salpingectomy, bilateral oophorectomy, or complete hysterectomy) or post-menopausal.

Women will be considered post-menopausal if they have been amenorrheic for 12 months without an alternative medical cause. The following age-specific requirements apply:

• Women < 50 years of age would be considered post-menopausal if they have been amenorrheic for 12 months or more following cessation of exogenous hormonal treatments and if they have luteinizing hormone and follicle-stimulating hormone levels in the post-menopausal range for the institution.

• Women ≥ 50 years of age would be considered post-menopausal if they have been amenorrheic for 12 months or more following cessation of all exogenous hormonal treatments, had radiation-induced menopause with last menses > 1 year ago, had chemotherapy-induced menopause with last menses > 1 year ago.

Highly effective methods of contraception, defined as one that results in a low failure rate (ie, less than 1% per year) when used consistently and correctly are described in Table 1. Note that some contraception methods are not considered highly effective (e.g. male or female condom with or without spermicide; female cap, diaphragm, or sponge with or without spermicide; non-copper containing intrauterine device; progestogen-only oral hormonal contraceptive pills where inhibition of ovulation is not the primary mode of action [excluding Cerazette/desogestrel which is considered highly effective]; and triphasic combined oral contraceptive pills).

Table 1. Highly Effective Methods of Contraception (<1% Failure Rate)

• Barrier/Intrauterine methods	• Hormonal Methods
• Copper T intrauterine device<br>• Levonorgestrel-releasing intrauterine system (e.g., Mirena®)[a]	• Implants: Etonogestrel-releasing implants: e.g. Implanon® or Norplant®<br>• Intravaginal: Ethinylestradiol/etonogestrel-releasing intravaginal devices: e.g. NuvaRing®<br>• Injection: Medroxyprogesterone injection: e.g. Depo-Provera®<br>• Combined Pill: Normal and low dose combined oral contraceptive pill<br>• Patch: Norelgestromin/ethinylestradiol-releasing transdermal system: e.g. Ortho Evra®<br>• Minipillc: Progesterone based oral contraceptive pill using desogestrel: Cerazette® is currently the only highly effective progesterone-based

8. STATISTICAL PLAN

The sample size for this phase 2 study is based on the following assumptions[26]:

The primary endpoint is progression-free survival.
The following hypotheses are used:

- p0=60%, assumed objective response after TACE or RFA as bridging therapy
- p1=85%, expected objective response after SBRT as bridging therapy

With a two-sided alpha value of 0.05 and an 80% power of detection of statistical significance, 28 patients were required. Assuming a dropout rate of about 20%, 35 patients were required for eligibility screening in this study.

Kaplan-Meier methods are used for survival analysis. Log-rank tests are performed for comparison of survival among different patient subgroups. Cox regression with univariable and multivariable analyses are performed for prognostic factors PFS and OS.

All statistical analyses are performed by Statistical Package for Social Sciences (SPSS) version 24, or R 3.6.1 or more advanced versions. Statistical significance is defined as *P* value < .05 (two-sided).

8.1 Analysis Endpoints

8.1.1 Efficacy Endpoint

Primary

- Progression-free survival (PFS)
    - PFS is calculated from the start of SBRT to the date of radiologically confirmed progressive disease based on RECIST 1.1,[23] mRECIST,[24] and PERCIST,[25] or death from any cause
- Best objective response as defined by RECIST 1.1,[23] mRECIST,[24] and PERCIST[25]

Secondary

- Local control rate as defined by RECIST 1.1,[23] mRECIST,[24] and PERCIST[25]
- Local control rate
- Overall survival (OS)
    - Calculated from start of SBRT to the date of death from any cause

8.1.2 Safety Endpoint

- Toxicity profiles as determined by Common Terminology Criteria for Adverse Events (CTCAE) version 4.0

8.1.3 Exploratory

- Prognostic factors of PFS and OS
- Predictive factors of objective response after SBRT

- Change in Child-Pugh scores before and after SBRT, especially the incidence of increase in Child-Pugh score by ≥2 points after SBRT

9. ETHICAL, REGULATORY & STUDY OVERSIGHT CONSIDERATIONS

9.1 Ethical Conduct of the Study

The study will be performed in accordance with ethical principles that have their origin in the Declaration of Helsinki and are consistent with ICH/Good Clinical Practice, and applicable regulatory requirements patient data protection.

9.2 Responsibilities of the Investigator(s)

The Investigator(s) undertake(s) to perform the Clinical Trial in accordance with this Clinical Trial Protocol, ICH Guideline for Good Clinical Practice (GCP) Guideline (ICH E6R2 Step 4) approved on November 9, 2016) and applicable regulatory requirements in Hong Kong. These documents state that the informed consent of the subjects is an essential precondition for participation in the clinical study.

9.3 Subject Information

An unconditional prerequisite for a patient participating in the study is his/her written informed consent. Adequate information must therefore be given to the subject by the Investigator(s) before informed consent is obtained. A person designated by the Investigator(s) may give the information, if permitted by local regulations. A subject information sheet in the local language and prepared in accordance with Good Clinical Practice will be provided by the Investigator(s) for the purpose of obtaining informed consent. In addition to this written information, the Investigator(s) or his/her/their designate will inform the subject verbally. In doing so, the wording used will be chosen so that the information can be fully and readily understood by laypersons.

The patient information sheet will be revised whenever important new information becomes available that may be relevant to the consent of patients.

9.4 Compensation to Subjects

The patients do not receive payment for taking part in this trial. Patients who are entitled to discounted or free health care, e.g. civil servants, will receive their entitled free treatment as normal.

9.5 Ethics Committee or Institutional Review Board

Prior to commencement of the study, the study protocol will be submitted together with its associated documents (patient information, informed consent forms,) to the institutional IRB for their favorable opinion. The favorable opinion/approval of the IRB will be filed in the study file. The study will only commence following provision of a written favorable opinion.

Any amendments to the protocol will be submitted to the IRB and they will be informed about SAEs in accordance with national and/or local requirements.

10. STUDY MANAGEMENT

10.1 Data Quality Assurance

The main objective is to obtain those data required by the study protocol in a complete, accurate, legible and timely fashion. The data in the Clinical Record Form (CRF) should be consistent with the relevant source documents.

The CRFs must be filled in completely and legibly (with either black or blue ballpoint pen, acceptable for use on official documents). Any amendments and corrections necessary must be undertaken and countersigned by the Investigator(s), stating the date of the amendment/correction. The Investigator(s) must state his/her reasons for the correction of important data. In the case of missing data/remarks, the entry spaces provided in the case report form should be cancelled out so as to avoid unnecessary follow-up inquiries.

The CRFs are regulatory documents and must be suitable for submission to authorities. According to the ICH guidelines for Good Clinical Practice, the Monitoring Team must check the CRF entries against source documents, except for the pre-identified source data directly recorded in the CRF. The Informed Consent Form will include a statement by which the patient allows the Sponsor’s duly authorized personnel, the Institutional Review Board/Ethics Committee (IRB/EC), and the regulatory authorities to have direct access to source data which support the data on the CRF. Such personnel, bound by professional secrecy, must keep confidential all personal identity or personal medical information (according to confidentiality rules).

10.2 Direct Access to Source Data/Documents

For the purpose of ensuring compliance with Clinical Trial Protocol, Good Clinical Practice and applicable regulatory requirements, the Investigator(s) should permit auditing by or on the behalf of the Sponsor and inspection by applicable regulatory authorities.

The Investigator(s) agree(s) to allow the auditors/inspectors to have direct access to the study records for review, being understood that this personnel is bound by professional secrecy, and as such will not disclose an personal identity or personal medical information.

The Investigator will make every effort to help with the performance of the audits and inspections, giving access to all necessary facilities, data, and documents.

As soon as the Investigator(s) is/are notified of a future inspection by the authorities, he will inform the Sponsor and authorize the Sponsor to participate in this inspection.

The confidentiality of the data verified and the protection of the patients should be respected during these inspections.

Any result and information arising from the inspections by the regulatory authorities will be immediately communicated by the Investigator(s) to the Sponsor.

The Investigator(s) shall take appropriate measures required by the Sponsor to take corrective actions for all problems found during the audit or inspections.

10.3 Study File, Database and Archiving

The Investigator(s) should have a study file for the study purpose. This file contains all relevant documents necessary for the conduct of the study. This file must be safely archived after termination of the study in accordance with the local relevant regulations.

APPENDIX 1. Prescription and Acceptance Criteria for SBRT (Modified from RTOG 1112 Protocol[27])

(I) Target Volume Delineation

The Gross Tumor Volume (GTV) is defined as all parenchymal and vascular HCC seen on MRI with gadoxetate disodium, and contrast-enhanced CT scan. Reference is also made with dual-tracer PET-CT scan images. GTV_P1 should represent the 'primary parenchymal (=P) dominant (=1)' GTV, upon which primary quality assurance will be based. Subsequent lesions can be labeled as GTV_P2, GTV_P3,).

MRI, dual-tracer PET-CT and contrast-enhanced CT images must be co-registered with the planning CT for every subject recruited into this study (tumor-to-tumor registration is highly recommended).

The Clinical Target Volume (CTV): For each GTV_P, the CTV is defined as the GTV (CTV_P1… CTV_P3), with no expansion. The minimal CTV_P is the GTV_P, with no expansion. It is expected that there will be no expansion from GTV to CTV for the majority of cases. Such CTVs may be treated to a microscopic dose (35 Gy) or up to as high as the prescription dose, at the investigator's discretion. Separate CTVs should be labeled CTV_P1, CTV_P2, and CTV_P3. The prescription dose should be annotated to each CTV after the final plan is complete (e.g. CTV_P1_50 for a 50Gy target, and CTV_P2_35 for a CTV treated to 35 Gy).

The Internal Target Volume (ITV): For each GTV_P or CTV_P, a margin may be added to account for the physiological motion of the tumors e.g. secondary to respiration or abdominal movements. The ITVs will be determined by the amplitude of the tumor and the liver based on 4D planning CT scans. For patients who will be treated with breath-hold technique, the ITV_P is the GTV_P with no expansion. Such ITV may be treated to a microscopic dose (35 Gy) or up to as high as the prescription dose, at the investigator's discretion. Separate ITVs should be labeled ITV_P1, ITV_P2, and ITV_P3. The prescription dose should be annotated to each ITV after the final plan is complete (e.g. ITV_P1_50 for a 50Gy target, and ITV_P2_35 for a ITV treated to 35 Gy).

The PTV will provide a margin around each ITVs to compensate for set-up. PTV nomenclature should follow GTV/CTV/ITV nomenclature guidelines. For example, PTV_P for the PTV around the ITV_P and PTV_P1 and PTV_P2 for PTVs around CTV_P1 and CTV_P2. A minimum PTV margin of 2–3 mm around each ITV is required in all directions. PTVs should not be manually modified due to proximity of adjacent OARs. The final PTVs should have dose annotated once the plan is final. Examples are PTV_P1_50 and PTV_P1_35 for targets treated to 50Gy and 35Gy, respectively.

(II) Critical Structures or Organs-at-Risk Delineation

The following critical normal structures (known as organs-at-risks [OAR] must be contoured by the investigator(s) for each patient:

Liver
Liver minus GTVs
Esophagus
Stomach
Duodenum
Small bowel
Large bowel
Gallbladder
Common bile duct
Spinal cord
Spina cord PRV5 (spinal cord + 5mm)
Right kidney
Left kidney
Skin
External chest wall
Heart
Ascites (if present, for treatment positional verification only though no constraint will be applied to treatment plan optimisation)
Subphrenic fat (if present, for treatment positional verification only though no constraint will be applied to treatment plan optimization)

(III) Prescription Dose

Absorbed dose: 35Gy to 50Gy in 5 fractions. The prescription dose may be 50Gy, 45Gy, 40Gy, or 35 Gy in 5 fractions, based on normal tissue constraints. The dose to multiple PTVs may be different. The goal is to use the highest allowable prescription dose to the primary target, while respecting normal tissue constraints. The minimal planned prescription dose to PTVs is 35Gy.

(IV) Dose Specifications

The prescription isodose should encompass 95% of PTV. The dose to multiple PTVs within the same patient may vary. If there are multiple PTVs, each should be planned for one of the prescription doses listed above, with each specific covering isodose planned to encompass 95% of each PTV, with normalization to the PTV receiving the highest dose. The highest allowable doses to the target volumes that maintain normal tissue constraints should be used. A goal is that 100% of the CTV is encompassed by the prescription dose. The unit of dose is Gy.

Dose prescription is based on the volume of normal tissues irradiated (correlated with mean liver dose), as well as proximity of stomach, duodenum, small and large bowel (GI luminal structures) to the target volumes, as normal tissue constraints must be maintained in this study.

In the absence of adjacent GI luminal structures that may limit dose, the PTV dose prescription should be as high as possible based on mean liver dose (MLD, defined as the mean dose to the liver minus all GTVs), with 4 potential dose levels: Use of effective liver volume (Veff) to aid in dose allocation is permitted but not mandatory. If there are discrepancies in the Veff and MLD for the prescription dose allocation, MLD has priority.

Priority Constraint	Optional Constraint	Prescription Dose	
Mean liver dose (liver minus all GTVs)	Liver Veff	Planned prescription	If the maximum allowed MLD is exceeded at this planned dose
13	< 25%	50	Reduce to 45 Gy and re-evaluate
15	25-29%	45	Reduce to 40 Gy and re-evaluate
15	30-34%	40	Reduce to 35 Gy and re-evaluate
15.5	35-44%	35	Do not treat; out of study

Compliance Criteria

The review process for this protocol is aimed at assuring correct contouring of target and critical structures, as well as appropriate SBRT planning. These reviews should avoid violations and deviations for this protocol. Each treatment shall be judged according to the protocol guidelines, with variations and deviations defined below:

<u>Total Treatment Duration</u>

Per protocol: All treatment falls within 14 calendar days
Variation Acceptable: All treatments fall within 16 days
Deviation Unacceptable: All treatments that take 16 or more calendar days to complete

<u>GTV Compliance</u>

Per protocol: no edits required
Variation acceptable: Variations in GTV or CTV other than deviation unacceptable
Deviation unacceptable: Definite HCC not contoured within GTV

<u>PTV Compliance</u>

PTV contouring per protocol: PTV > 4 mm and < 20 mm
Variation acceptable: PTV 3-4 mm or 20-25 mm
Deviation unacceptable PTV < 3 mm or > 25 mm

<u>PTV Dosimetry</u>

Target coverage for each PTV should be considered on its own. If there are multiple tumors, the primary (dominant) PTV should be labeled #1. The intent is for prescription dose to cover 95% of each PTV. If PTVs are not treated as per guidelines, this is a deviation unacceptable. The PTV should be treated to as high a dose as possible, respecting normal tissue constraints (as mentioned below), as a dose response has been observed. Modifying required PTVs due to close proximity of adjacent OARs is not permitted.

The following table describes variations and deviations in the prescription dose (dose covering 95% of the PTV). Treating “per protocol” should always be the planning intent.

**Dsoe to 95% PTV**	**PTVs around GTVs ***	**PTVs around non-GTV ITVs/CTVs ***
Per protocol	Prescription dose +/- 5%	Prescription dose +/- 5%
Variation acceptable	90-95% or 105-110% of prescription dose, and ≥ 35Gy	85-95% or 105-115% of prescription dose and ≥ 35Gy
Deviation unacceptable	< 90% or > 110% of prescription dose, or < 32Gy	< 85% or >115% of prescription dose, or < 32Gy
Overall plan deviation unacceptable	< 32Gy	

*Note that lower doses than the dose-allocation schedule are acceptable if they are required due to adjacent GI luminal structures that may limit the deliverable dose.

Compliance for Critical Structures (organs at risk, OARs) If non-hepatic OARs limit the prescription dose, the highest dose (from the 4 prescription doses) should be used, while maintaining OAR dose constraints.

**Prescription dose**	**Liver (minus GTV) mean dose**		
	Per protocol	Variation acceptable	Deviation unacceptable
50Gy	≤ 13Gy	13-13.2Gy	> 13.2Gy
45Gy	≤ 15Gy	15-15.2Gy	> 15.2Gy
40Gy	≤ 15Gy	15-15.2Gy	> 15.2Gy
35Gy	≤ 15.5Gy	15.5-15.7Gy	> 15.7Gy

**Non-liver OARs**	**Per protocol**	**Variation acceptable**	**Deviation unacceptable**
Eesophagus max (to 0.5cc)	43Gy	> 43 but ≤ 45Gy	> 45Gy
Stomach max (to 0.5cc)	43Gy	> 43 but ≤ 45Gy	> 45Gy
Duodenum max (to 0.5cc)	43Gy	> 43 but ≤ 45Gy	> 45Gy
Small bowel (to 0.5cc)	43Gy	> 43 but ≤ 45Gy	> 45Gy
Large bowel max (to 0.5cc)	48Gy	> 48 but ≤ 50Gy	> 50Gy
Spinal cord + 5mm max (0.5cc)	25Gy	> 25 but ≤ 28Gy	> 28Gy
Kidneys: bilateral mean dose	≤ 10Gy	> 10 but ≤ 12Gy	> 12Gy

The following organ dose constraints are guidelines, not mandatory:
Liver minus all GTVs: > 700cc and V10Gy < 70%
Heart max (30cc): < 30Gy
Great vessel max (0.5cc): < 60Gy
Skin (external) max (0.5cc): < 32Gy
Chest wall max (0.5cc): < 50Gy
Gallbladder max (0.5cc): < 5Gy
Common bile duct max (0.5cc): < 50Gy (even though the bile duct is not often well visualized, it is always within the portal region and may be within high dose volumes for central targets, so efforts to reduce hot spots in this region are warranted)

APPENDIX 2. Scanning algorithm of magnetic resonance imaging (MRI) scan with gadoxetate disodium injection

MRI scanning was conducted with a 3-Tesla MR scanner (Philips Achieva 3.0T TX, Philips Medical Systems, Best, The Netherlands) throughout the whole study as shown below. The same scanner is used for all scans for all patients recruited into this study. MRI scans were performed at baseline, then at every 3 months until liver transplantation was performed or progressive disease was observed, whichever came earlier. All HCC lesions were evaluated by two independent radiologists and any discrepancy in deteremining the nature of the lesions was solved by consensus.

(1) T2-weighted TSE single-shot in 5mm section thickness

(2) Breath-hold triggered T1-weighted in-phase and opposed sequences in 7mm section thickness

(3) T2-weighted SPAIR with TE 80 and 160 in 7mm section thickness

(4) Diffusion-weighted single-shot echo planar imaging with respiratory triggering

(5) Gadoxetate disodium (0.025mmol/kg body weight)-enhanced dynamic phases: unenhanced, late arterial-phase, portal-venous phase, and delayed phases with T1-weighted three-dimensional (3D) turbo-field-echo sequence (T1 high-resolution isotropic volume examination, THRIVE) with a 2-mm section thickness and a FOV of 32-38 cm.

APPENDIX 3. Positron-emission tomography with integrated contrast-enhanced computed tomography (PET-CT) with two radiotracers (11C-acetate [ACC] and 18F-fluorodeoxyglucose [FDG])

Dual Tracer PET-CT scan was be performed at baseline, and then every 3 months after SBRT until liver transplantation done or until progressive disease whichever came earlier. All scans were performed using the same scanner and protocol (Discovery VCT, 64-MDCT; GE Healthcare Bio-Sciences Corp., Piscataway, New Jersey, USA). All patients are required to fast for ≥6 hours, and the blood glucose concentration is ≤ 8 mmol/L before radiopharmaceutical injection. ACC (440–590 MBq; 8.0 MBq/kg of body weight) was then administered intravenously, and limited upper abdomen coverage acquired at 1 minute followed by a whole body imaging is performed at 11 minutes after injection. Data acquisition with an integrated in-line PET-CT scanner (Discovery VCT, 64-multislice CT; GE Healthcare Bio-Sciences Corp, NJ) begins with non-contrast whole-body CT for attenuation correction, followed by PET with a 2-minute emission acquisition time and a 16.2-cm axial field of view per position. At 45 minutes after ACC injection, 330-520 MBq (6.3 MBq/kg of body weight) FDG was injected and the same image acquisition was obtained at 60 minutes after FDG administration. Enhanced (with Omnipaque 300, 2ml/kg; GE Healthcare) CT scanning with an in-house 3-phase (early and late arterial and porto-venous) contrast-enhanced protocol covering the whole abdomen was then performed for additional diagnostic evaluation. All HCC lesions were evaluated by two independent radiologists and any discrepancy in deteremining the nature of the lesions was solved by consensus.

Determination of MTV and TLG/TLA

Firstly, spherical volume of interests (VOI) with a diameter of 3cm were positioned in the right lobe of liver at least 3 cm away from the tumors (if present) or the mediastinal blood pool if the VOI cannot be reliably placed in the liver without being interfered by the adjacent tumor lesions to determine the background standardized uptake value (SUV) as reference.[25] The mean SUV and standard deviation (SD) of these VOIs based on FDG and ACC were calculated and recorded as SUV_{ref} and SD_{ref} respectively. For those patients whose lesion was seen with only 1 type of radiotracer, the same ROI was applied at the same level on the axial images of the other radiotracer, correcting for any misalignment or misregistration of present. Background threshold method was used to determine the threshold for subsequent delineation of the MTV as follows: Threshold = $SUV_{ref} + 2SD_{ref}$. TLG with respect to FDG or TLA with respect to ACC of any tumor lesions is the product of SUVmean and MTV of that lesion with respect to each radiotracer. Only lesions that could be delineated using the threshold were deemed reportable and were further measured using automatic segmentation using a GE advantage workstation. SUVmax, MTV and TLG/TLA were then computed. Lesions with metabolic activities less than these threshold will be considered as “non-reportable”, and therefore “normal”. All these parameters will be recorded as zero.

APPENDIX 4. RECIST 1.1 and mRECIST

**RECIST 1.1**	**mRECIST**
CR – Disappearance of all target lesions	CR – Disappearance of any intratumoral arterial enhancement in all target lesions
PR – At least a 30% decrease in the sum of diameters of target lesions, taking as reference the baseline sum of the diameters of target lesions	PR – At least a 30% decrease in the sum of diameters of viable (enhancement in the arterial phase) target lesions, taking as reference the baseline sum of the diameters of target lesions
SD – Any cases that do not qualify for either partial response or progressive disease	SD – Any cases that do not qualify for either partial response or progressive disease
PD – An increase of at least 20% in the sum of the diameters of target lesions, taking as reference the smallest sum of the diameters of target lesions recorded since treatment started	PD – An increase of at least 20% in the sum of the diameters of viable (enhancing) target lesions, taking as reference the smallest sum of the diameters of viable (enhancing) target lesions recorded since treatment started

CR, complete response; PD, progressive disease; PR, partial response; SD, stable disease.

APPENDIX 5. PERCIST 1.0

	PERCIST 1.0
	1. Measurable target lesion in hottest single tumor lesion SUL of "maximal 1.2 cm diameter volume ROI in tumor" (SUL peak). SUL peak is at least 1.5 fold greater than liver SUL mean + 2 SDs (in 3cm spherical ROI in normal right lobe of liver). If liver is abnormal, primary tumor should have uptake > 2.0 × SUL mean of blood pool in 1cm diameter ROI in descending thoracic aorta extended over 2cm z-axis..<br>2. Tumor with maximal SUL peak is assessed after treatment. Although typically this is in same region of tumor as that with highest SUL peak at baseline, it need not be.
	3. Uptake measurements should be made for peak and maximal single-voxel tumor SUL. Other SUV metrics, including SUL mean at 50% or 70% of SUV peak, can be collected as exploratory data; TLG/TLA can be collected ideally on basis of voxels more intense than 2SDs above liver mean SUL (see below).
	4. These parameters can be recorded as exploratory data on up to 5 measurable target lesions, typically the 5 hottest lesions, which are typically the largest, and no more than 2 per organ. Tumor size of these lesions can be determined per RECIST 1.1.
Normalisation of uptake	Normal liver SUL must be within 20% (and, 0.3 SUL mean units) for baseline and follow-up study to be assessable. If liver is abnormal, blood pool SUL must be within 20% (and 0.3 SUL mean units) for baseline and follow-up study to be assessable. Uptake time of baseline study and follow-up study 2 must be within 15 minutes of each other to be assessable. Typically, these are at mean of 60 minutes after injection but no less than 50 minutes after injection. Same scanner, or same scanner model at same site, injected

	dose, acquisition protocol (2- *v* 3-dimensional), and software for reconstruction, should be used. Scanners should provide reproducible data and be properly calibrated.
Objective response	CMR – complete resolution of FDG and ACC uptake within measurable target lesion so that it is less than mean liver activity and indistinguishable from surrounding background blood-pool levels. Disappearance of all other lesions to background blood pool levels. Percentage decline in SUL should be recorded from measurable region, as well as (ideally) time in weeks after treatment was begun (i.e., CMR-90, 4). No new FDG–avid lesions in pattern typical of cancer. If progression by RECIST, must verify with follow-up.
	PMR – reduction of minimum of 30% in target measurable tumor ACC and/or FDG SUL peak. Absolute drop in SUL must be at least 0.8 SUL units, as well. Measurement is commonly in same lesion as baseline but can be another lesion if that lesion was previously present and is the most active lesion after treatment. ROI does not have to be in precisely same area as baseline scan, though typically it is. No increase, > 30% in SUL or size of target or nontarget lesions (i.e., no PD by RECIST or IWC) (if PD anatomically, must verify with follow-up). Reduction in extent of tumor ACC and FDG uptake is not requirement for PMR. Percentage decline in SUL should be recorded, as well as (ideally) time in weeks after treatment was begun (i.e., PMR-40, 3). No new lesions.
	SMD – not CMR, PMR, or PMD. SUL peak in metabolic target lesion should be recorded, as well as (ideally) time from start of most recent therapy, in weeks (i.e., SMD -15, 7).
	PMD – > 30% increase in ACC and/or FDG SUL peak, with > 0.8 SUL unit increase in tumor SUV peak from baseline scan in pattern typical of tumor and not of infection/treatment effect. OR: Visible increase in extent of ACC and/or FDG tumor

	uptake (75% in TLA/TLG volume with no decline in SUL. OR: New ACC and/or FDG–avid lesions that are typical of cancer and not related to treatment effect or infection. PMD other than new visceral lesions should be confirmed on follow-up study within 1 month unless PMD also is clearly associated with progressive disease by RECIST 1.1. PMD should be reported to include percentage change in SUV peak, (ideally, time after treatment, in weeks) and whether new lesions are present/absent and their number (i.e., PMD, +35, 4, new: 5). Because SUL is continuous variable, dividing response criteria into limited number of somewhat arbitrary response categories loses much data. For this reason, PERCIST preserves percentage declines in SUV peak in each reported category. Because rapidity with which scan normalizes is important (faster appears better), PERCIST asks for time from start of treatment as part of reporting. For example, CMR 90, 1, is probably superior to CMR 90, 10, especially if latter patient were SMD20, 1. More than one measurement of PET response may be needed at differing times, and it may be treatment type–dependent. PERCIST 1.0 evaluates SUL peak of only hottest tumor. This is possible limitation of approach, but lesions and their responses are highly correlated in general. Additional data are required to determine how many lesions should be assessed over 1. A suggested option is to include the 5 hottest lesions, or the 5 observed on RECIST 1.1 that are most measurable. Percentage change in SUL can be reported for single lesion with largest increase in uptake or smallest decline in uptake. Additional studies will be needed to define how many lesions are optimal for assessment.

CMR, complete metabolic response; PMR, partial metabolic response; PD, progressive disease; SMD, stable metabolic disease; PMD, progressive metabolic disease; CR, complete remission; PR, partial remission; NC, no change, SUL, standardized uptake value corrected for lean body mass.

For PERCIST: Single-voxel SUL is commonly used but has been reported to be less reproducible than SUL peak, especially with very small single-voxel values. It is suggested, but not required, that lesions assessed on PERCIST be larger than the 1.5-cm-diameter volume ROI used to minimise partial-volume effects. Percentage changes are proposed to deal with SUL peak changes. Use of maximal SUL could be explored. If 5 lesions are used as exploratory approach, it is suggested that sum of SULs of baseline 5 lesions serve as baseline for study. After treatment, sum of same 5 lesions should be used. Percentage change in SUL is based on change in these sums from study 1 to study 2. Exploratory analysis can include calculating percentage change in SUL in individual lesions and averaging them. This may produce different result. We believe summed SUL approach will be less prone to minor errors in measurements. For total lesion glycolysis: Exploratory analysis can include either all foci of tumor with maximal SUL. 2SDs above normal liver, 5 lesions with highest SUL, or lesion with highest SUL. It is suggested that threshold approach, typically at 2 SDs above normal liver SUL, be used to generate lower bounds of ROI (3SDs could be used for very active tumors). We believe this approach will be less variable than methods based on maximal SUL with percentage of maximal cutoff. Criteria for progression include 75% growth in TLG/TLA for SUL and are conservatively placed at 75% increase. Because 20% increase in EORTC linear size scales to 73% volume increase, the figures are comparable. Progression is judged from best response if being assessed after first scan was performed. For response by TLG/TLA, we propose 45% reduction as useful starting point, but more data are needed to make firm recommendations. If TLG/TLA is determined, explicit methodologic details should be provided. It should not be a primary metric, but a secondary endpoint at this time.

REFERENCES

1. Bray F, Ferlay J, Soerjomataram I, et al. Global cancer statistics 2018: GLOBOCAN estimates of incidence and mortality worldwide for 36 cancers in 185 countries. *CA Cancer J Clin.* 2018;68(6):394-424.
2. Top Ten Cancers. Hong Kong Cancer Registry. https://www3.ha.org.hk/cancereg/topten.html.
3. Mazzaferro V, Regalia E, Doci R, et al. Liver transplantation for the treatment of small hepatocellular carcinomas in patients with cirrhosis. *N Engl J Med.* 1996;334:693-699.
4. Yao F, Ferrell L, Bass N, et al. Liver transplantation for hepatocellular carcinoma: expansion of the tumor size limits does not adversely impact survival. *Hepatology.* 2001;33(6):1394-1403.
5. Pompili M, Francica G, Ponziani F, et al. Bridging and downstaging treatments for hepatocellular carcinoma in patients on the waiting list for liver transplantation. *World J Gastroenterol.* 2013;19(43):7515-7530.
6. Chok KS, Cheung TT, Lo RC, et al. Pilot study of high-intensity focused ultrasound ablation as a bridging therapy for hepatocellular carcinoma patients wait-listed for liver transplantation. *Liver Transpl.* 2014;20(8):912-921.
7. Mohamed M, Katz AW, Tejani MA, et al. Comparison of outcomes between SBRT, yttrium-90 radioembolization, transarterial chemoembolization, and radiofrequency ablation as bridge to transplant for hepatocellular carcinoma. *Adv Radiat Oncol.* 2015;1(1):35-42.
8. O'Connor JK, Trotter J, Davis GL, et al. Long-term outcomes of stereotactic body radiation therapy in the treatment of hepatocellular cancer as a bridge to transplantation. *Liver Transpl.* 2012;18(8):949-954.
9. Mazloom A, Hezel AF, Katz AW. Stereotactic body radiation therapy as a bridge to transplantation and for recurrent disease in the transplanted liver of a patient with hepatocellular carcinoma. *Case Rep Oncol.* 2014;7(1):18-22.
10. Guarneri A, Franco P, Romagnoli R, et al. Stereotactic ablative radiation therapy prior to liver transplantation in hepatocellular carcinoma. *Radiol Med.* 2016;121(11):873-881.
11. Sapisochin G, Barry A, Doherty M, et al. Stereotactic body radiotherapy vs. TACE or RFA as a bridge to transplant in patients with hepatocellular carcinoma. An intention-to-treat analysis. *J Hepatol.* 2017;67(1):92-99.
12. Mannina EM, Cardenes HR, Lasley FD, et al. Role of stereotactic body radiation therapy before orthotopic liver transplantation: retrospective evaluation of pathologic response and outcomes. *Int J Radiat Oncol Biol Phys.* 2017;97(5):931-938.
13. Moore A, Cohen-Naftaly M, Tobar A, et al. Stereotactic body radiation therapy (SBRT) for definitive treatment and as a bridge to liver transplantation in early stage inoperable Hepatocellular carcinoma. *Radiat Oncol.* 2017;12(1):163.

14. Gresswell S, Tobillo R, Hasan S, et al. Stereotactic body radiotherapy used as a bridge to liver transplant in patients with hepatocellular carcinoma and Child-Pugh score ≥8 cirrhosis. *J Radiosurg SBRT*. 2018;5(4):261-267.

15. Uemura T, Kirichenko A, Bunker M, et al. Stereotactic Body Radiation Therapy: A new strategy for loco-regional treatment for hepatocellular carcinoma while awaiting liver transplantation. *World J Surg*. 2019;43(3):886-893.

16. Choi JY, Lee JM, Sirlin CB. CT and MR imaging diagnosis and staging of hepatocellular carcinoma: part II. Extracellular agents, hepatobiliary agents, and ancillary imaging features. *Radiology*. 2014;273:30-50.

17. Lee DH, Lee JM, Baek JH, et al. Diagnostic performance of gadoxetic acid-enhanced liver MR imaging in the detection of HCCs and allocation of transplant recipients on the basis of the Milan criteria and UNOS guidelines: correlation with histopathologic findings. *Radiology*. 2015;274(1):149-160.

18. Li J, Wang J, Lei L, et al. The diagnostic performance of gadoxetic acid disodium-enhanced magnetic resonance imaging and contrast-enhanced multi-detector computed tomography in detecting hepatocellular carcinoma: a meta-analysis of eight prospective studies. *Eur Radiol*. 2019; 29(12):6519-6528.

19. Duncan KJ, Ma N, Vreugdenburg TD, et al. Gadoxetic acid-enhanced MRI for the characterization of hepatocellular carcinoma: a systematic review and meta-analysis. *J Magn Reson Imaging*. 2017;45(1):281-290.

20. Ho CL, Yu SC, Yeung DW. 11C-acetate Pet imaging in hepatocellular carcinoma and other liver masses. *J Nucl Med*. 2003;44(2):213-221.

21. Park JW, Kim JH, Kim SK, et al. A prospective evaluation of 18F-FDG and 11C-acetate PET/CT for detection of primary and metastatic hepatocellular carcinoma. *J Nucl Med*. 2008;49(12):1912-1921.

22. Cheung TT, Ho CL, Lo CM, et al. 11C-acetate and 18F-FDG PET/CT for clinical staging and selection of patients with hepatocellular carcinoma for liver transplantation on the basis of Milan criteria: surgeon's perspective. *J Nucl Med*. 2013;54(2):192-200.

23. Eisenhauer EA, Therasse P, Bogaert J, et al. New response evaluation criteria in solid tumours: Revised RECIST guideline (version 1.1). *Eur J Cancer*. 2009;45(2):228-247.

24. Lencioni R, Llovet JM. Modified RECIST (mRECIST) assessment for hepatocellular carcinoma. *Semin Liver Dis*. 2010;30(1):52-60.

25. Wahl RL, Jacene H, Kasamon Y, et al. From RECIST to PERCIST: Evolving Considerations for PET response criteria in solid tumors. *J Nucl Med*. 2009;50 Suppl 1(Suppl 1):122S-150S.

26. Kwak M, Jung SH. Phase II clinical trials with time-to-event endpoints: optimal two-stage designs with one-sample log-rank test. *Stat Med*. 2014;33(12):2004-2016.

27 . RTOG-1112 Randomized Phase III Study of Sorafenib versus Stereotactic Body Radiation Therapy followed by Sorafenib in Hepatocellular Carcinoma. NRG Oncology. https://www.nrgoncology.org/Clinical-Trials/Protocol/rtog-1112?filter=rtog-1112.
