# Supplementary material for: Stereotactic Body Radiotherapy and Liver Transplant for Liver Cancer: A Nonrandomized Controlled Trial
Source: JAMA Netw Open. 2024 Jun 10;7(6):e2415998. doi: 10.1001/jamanetworkopen.2024.15998 (PMC11165380; doi:10.1001/jamanetworkopen.2024.15998)
Supplement: Supplement 2. — eFigure 1. Dual-Tracer PET-CT Images With Dual Tracers (11C-Acetate [ACC] and 18F-Deoxyglucose [FDG]) in a 60-Year-Old Patient With Two Hepatocellular Carcinoma Lesions in (A) Segment 4b (SUVmax 3.6 With ACC) and (B) Segment 6 (SUVmax 2.9 With ACC) Before (Indicated by Arrows) and After Stereotactic Body Radiation Therapy (SBRT) eFigure 2. Formalin-Fixed Paraffin-Embedded Sections Under Microscopy in (A) Low-Power Field, and (B) High-Power Field Demonstrating Pathological Complete Response of a Hepatocellular Carcinoma Organized by a Fibrous Capsule in the Background of Cirrhosis Following Stereotactic Body Radiation Therapy eFigure 3. (A) Progression-Free Survival of Patients Who Had Complete Metabolic Response (CMR) and Those Who Did Not Have CMR Defined by PERCIST After Stereotactic Body Radiation Therapy and (B) Overall Survival of Patients Who Had Complete Metabolic Response (CMR) and Those Who Did Not Have CMR Defined by PERCIST After Stereotactic Body Radiation Therapy eFigure 4. (A) Progression-Free Survival of Patients Whose Hepatocellular Carcinoma Were Within the Milan Criteria and Beyond the Milan Criteria and (B) Overall Survival of Patients Whose Hepatocellular Carcinoma Were Within the Milan Criteria and Beyond the Milan Critieria eFigure 5. (A) Progression-Free Survival of Patients Who Received and Did Not Receive Liver Transplantation After Stereotactic Body Radiation Therapy and (B) Overall Survival of Patients Who Received and Did Not Receive Liver Transplantation After Stereotactic Body Radiation Therapy eTable 1. Dose Parameters of Gross Tumor Volumes and Planning Target Volumes of All Hepatocellular Carcinoma Lesions Treated With Stereotactic Body Radiation Therapy eTable 2. Dose Parameters of All Important Organs-at-Risk of All Patients eTable 3. Child-Pugh Scores Before and After Stereotactic Body Radiation Therapy (N = 32) eTable 4. Change in Child-Pugh Scores After Stereotactic Body Radiation Therapy (N = 32) eTable 5. Number of Patients Wh [file jamanetwopen-e2415998-s002.pdf]

Supplementary Online Content

Lee VHF, Vardhanabhuti V, Wong TCL, et al. Stereotactic body radiotherapy and liver transplant for liver cancer: a nonrandomized controlled trial. *JAMA Netw Open.* 2024;7(6):e2415998. doi:10.1001/jamanetworkopen.2024.15998

eFigure 1. Dual-Tracer PET-CT Images With Dual Tracers (11C-Acetate [ACC] and 18F-Deoxyglucose [FDG]) in a 60-Year-Old Patient With Two Hepatocellular Carcinoma Lesions in (A) Segment 4b (SUVmax 3.6 With ACC) and (B) Segment 6 (SUVmax 2.9 With ACC) Before (Indicated by Arrows) and After Stereotactic Body Radiation Therapy (SBRT)
eFigure 2. Formalin-Fixed Paraffin-Embedded Sections Under Microscopy in (A) Low-Power Field, and (B) High-Power Field Demonstrating Pathological Complete Response of a Hepatocellular Carcinoma Organized by a Fibrous Capsule in the Background of Cirrhosis Following Stereotactic Body Radiation Therapy
eFigure 3. (A) Progression-Free Survival of Patients Who Had Complete Metabolic Response (CMR) and Those Who Did Not Have CMR Defined by PERCIST After Stereotactic Body Radiation Therapy and (B) Overall Survival of Patients Who Had Complete Metabolic Response (CMR) and Those Who Did Not Have CMR Defined by PERCIST After Stereotactic Body Radiation Therapy
eFigure 4. (A) Progression-Free Survival of Patients Whose Hepatocellular Carcinoma Were Within the Milan Criteria and Beyond the Milan Criteria and (B) Overall Survival of Patients Whose Hepatocellular Carcinoma Were Within the Milan Criteria and Beyond the Milan Critieria
eFigure 5. (A) Progression-Free Survival of Patients Who Received and Did Not Receive Liver Transplantation After Stereotactic Body Radiation Therapy and (B) Overall Survival of Patients Who Received and Did Not Receive Liver Transplantation After Stereotactic Body Radiation Therapy
eTable 1. Dose Parameters of Gross Tumor Volumes and Planning Target Volumes of All Hepatocellular Carcinoma Lesions Treated With Stereotactic Body Radiation Therapy
eTable 2. Dose Parameters of All Important Organs-at-Risk of All Patients
eTable 3. Child-Pugh Scores Before and After Stereotactic Body Radiation Therapy (N = 32)
eTable 4. Change in Child-Pugh Scores After Stereotactic Body Radiation Therapy (N = 32)
eTable 5. Number of Patients Whose Pretreatment Parameters Are Beyond RTOG 1112 Trial Eligibility Criteria
eTable 6. Number of Patients Whose Dosimetric Parameters of Organs-at-Risk in Their SBRT Plans Beyond RTOG 1112 Trial Acceptance Criteria

This supplementary material has been provided by the authors to give readers additional information about their work.

© 2024 Lee VHF et al. *JAMA Network Open.*

eFigure 1. Dual-Tracer PET-CT Images With Dual Tracers (11C-Acetate [ACC] and 18F-Deoxyglucose [FDG]) in a 60-Year-Old Patient With Two Hepatocellular Carcinoma Lesions in (A) Segment 4b (SUVmax 3.6 With ACC) and (B) Segment 6 (SUVmax 2.9 With ACC) Before (Indicated by Arrows) and After Stereotactic Body Radiation Therapy (SBRT). Both lesions gradually showed complete metabolic response based on ACC at 3, 6, and 9 months after SBRT, but remained eumetabolic with FDG all along. Both lesions exhibited pathological complete necrosis in the liver explant following liver transplantation performed 16 months after SBRT.

(A)

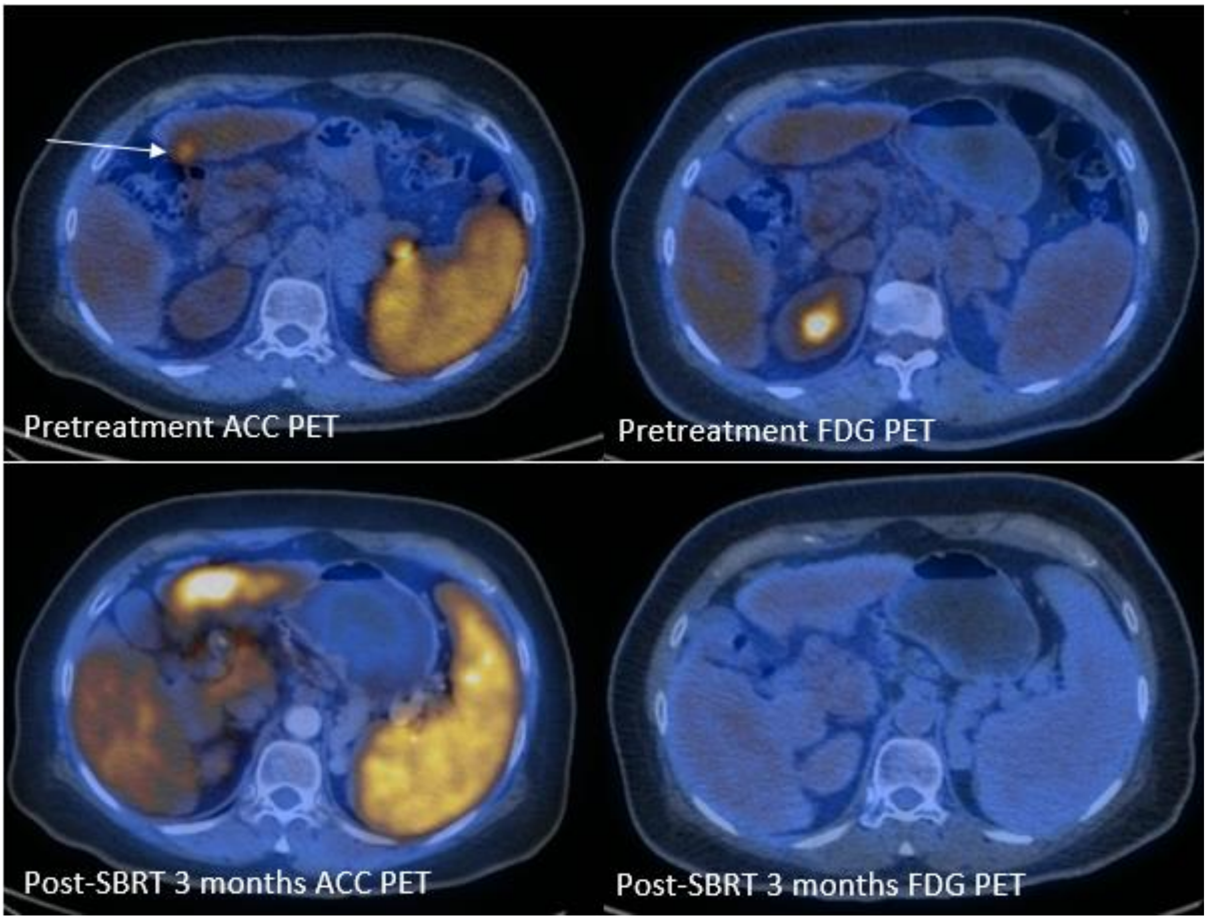

© 2024 Lee VHF et al. *JAMA Network Open.*

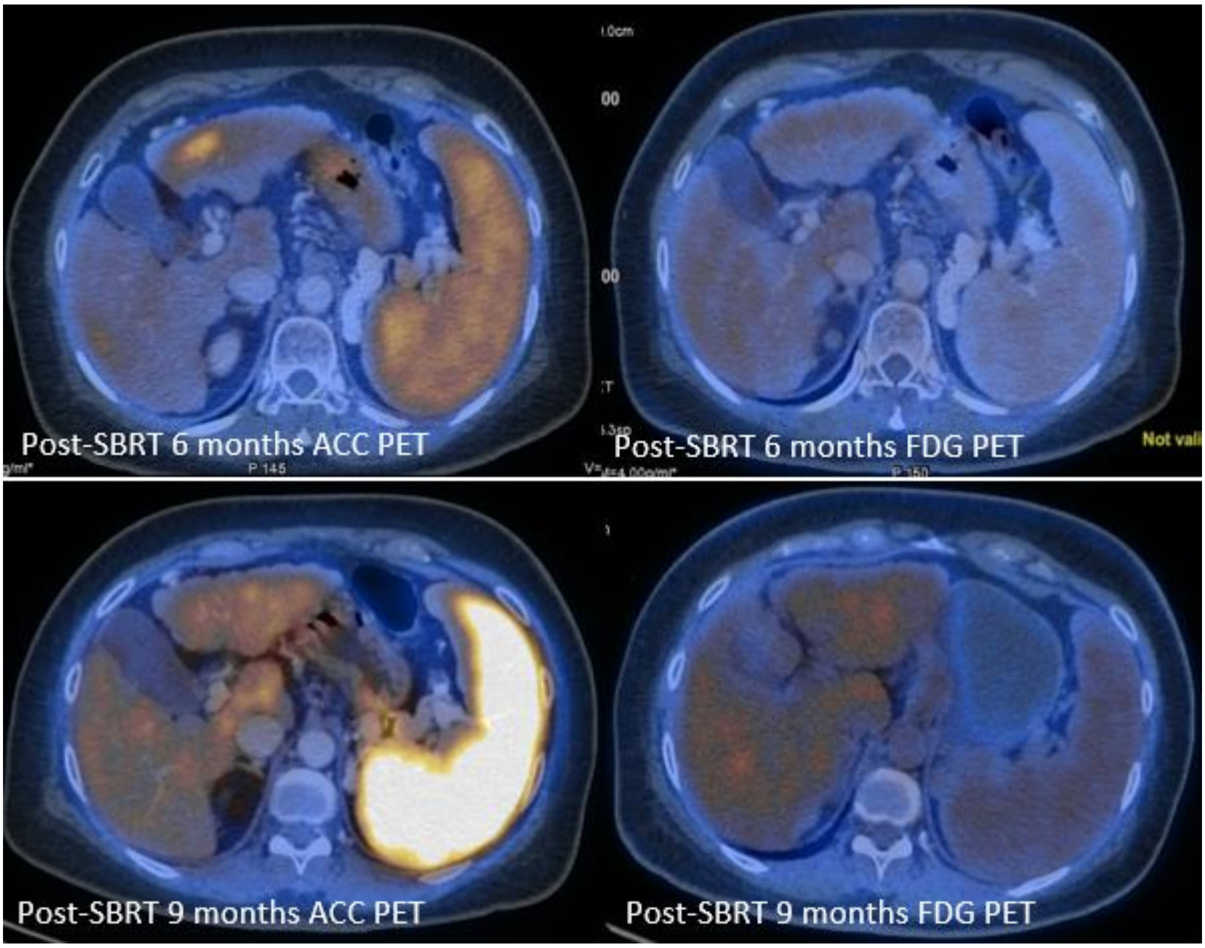

© 2024 Lee VHF et al. *JAMA Network Open.*

(B)

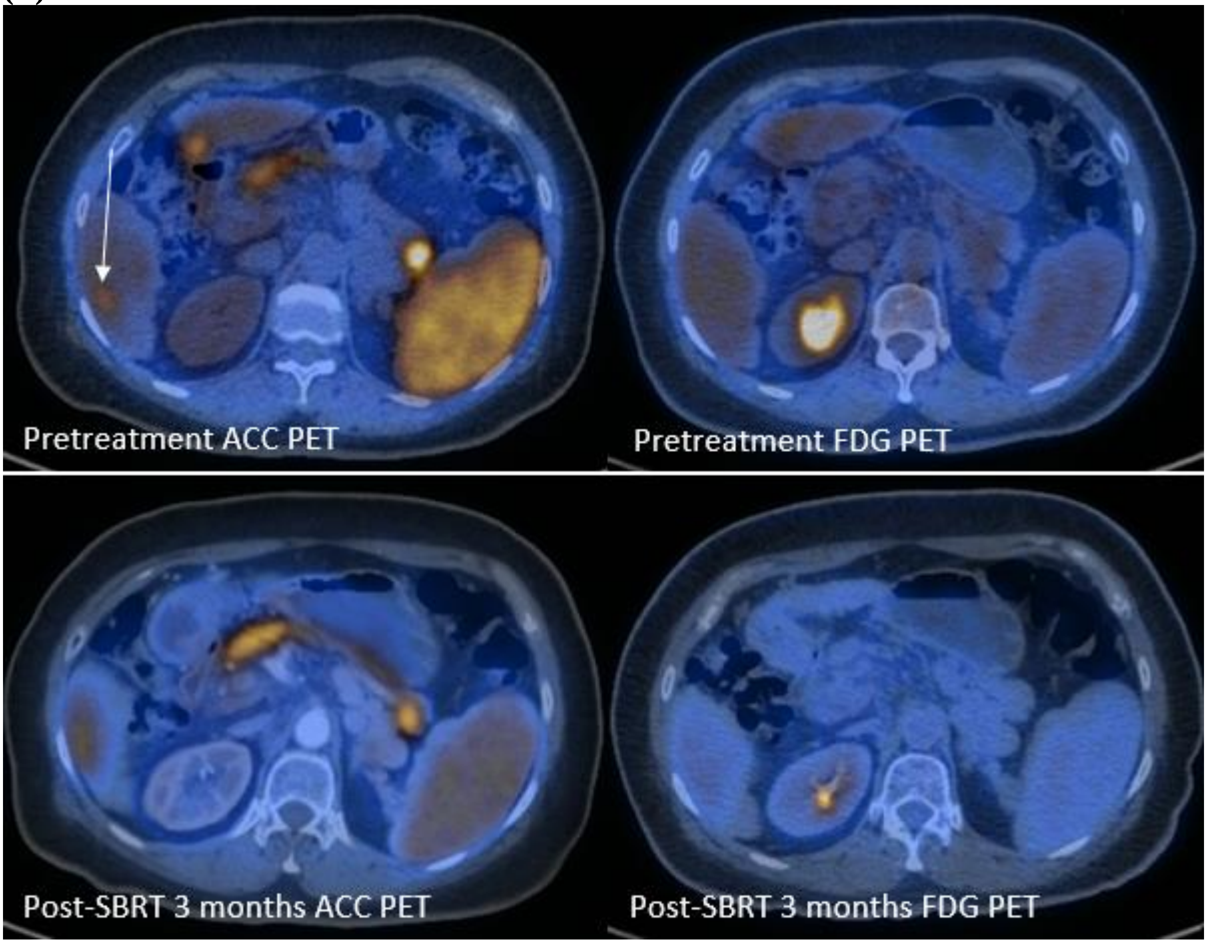

© 2024 Lee VHF et al. *JAMA Network Open.*

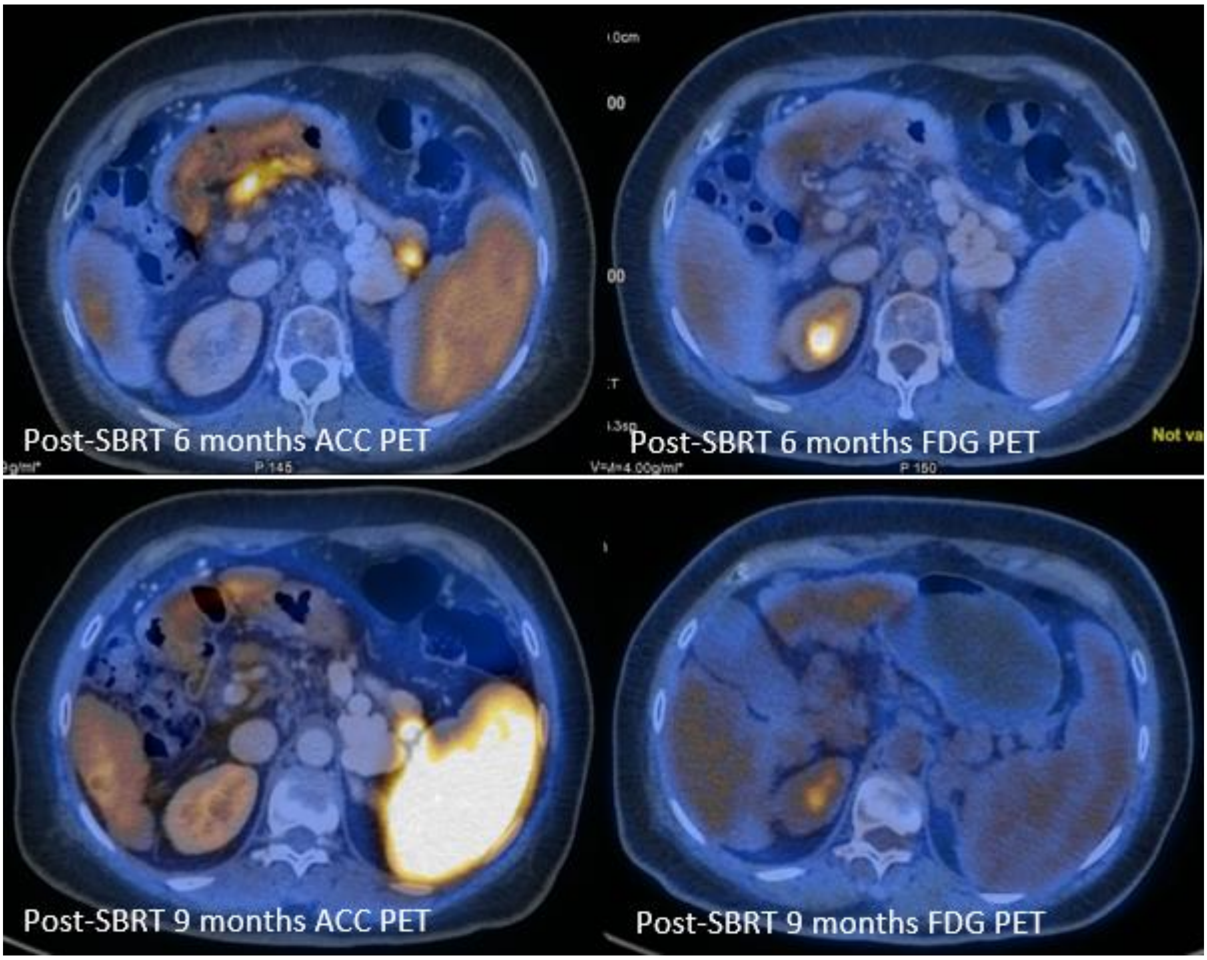

© 2024 Lee VHF et al. *JAMA Network Open.*

eFigure 2. Formalin-Fixed Paraffin-Embedded Sections Under Microscopy in (A) Low-Power Field, and (B) High-Power Field Demonstrating Pathological Complete Response of a Hepatocellular Carcinoma Organized by a Fibrous Capsule in the Background of Cirrhosis Following Stereotactic Body Radiation Therapy

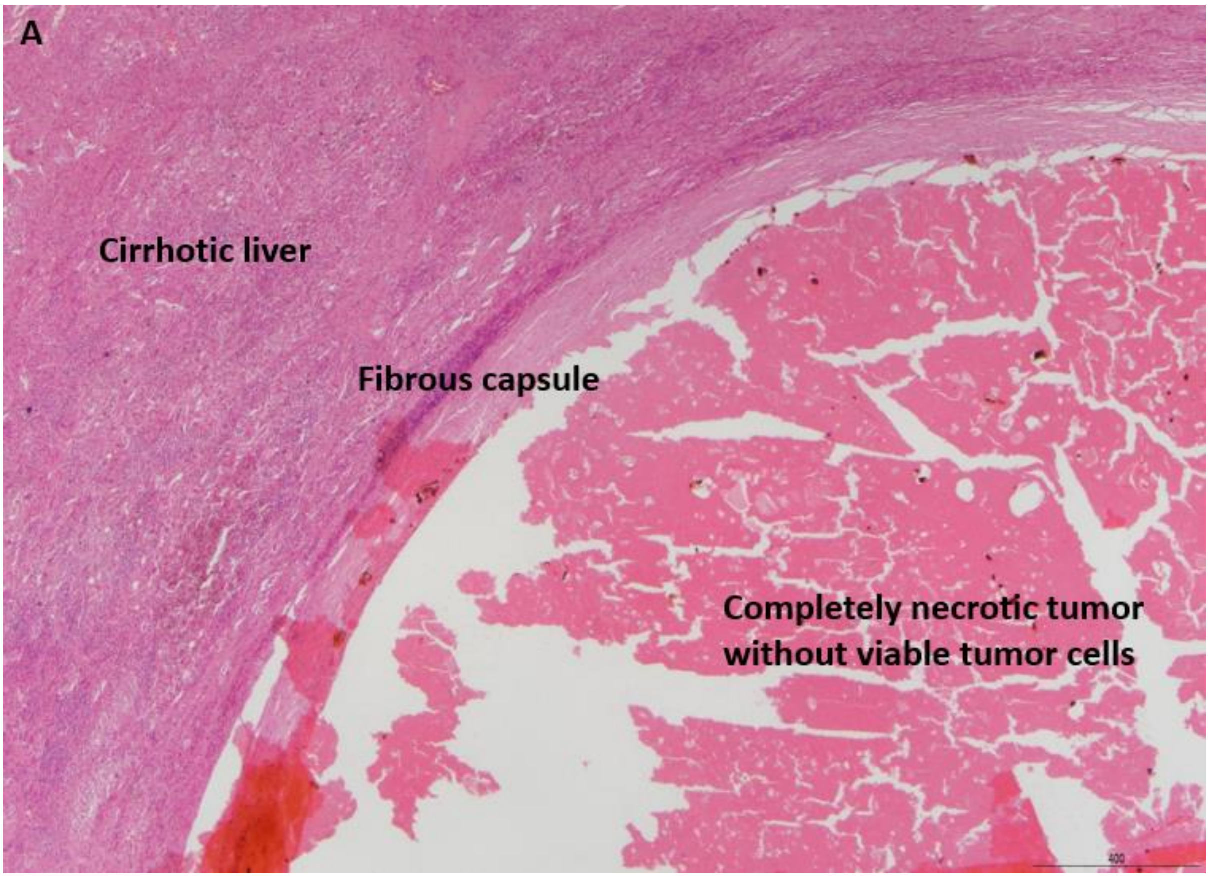

© 2024 Lee VHF et al. *JAMA Network Open.*

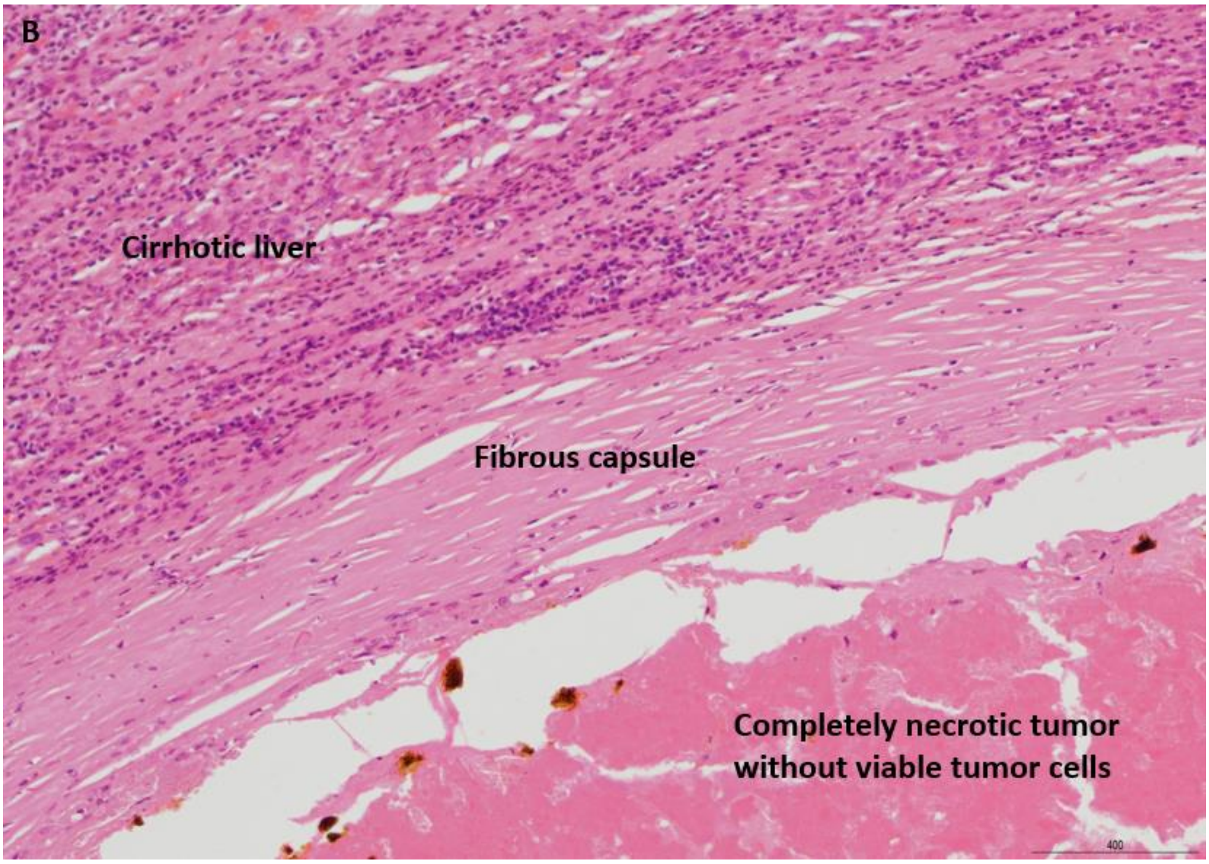

© 2024 Lee VHF et al. *JAMA Network Open.*

eFigure 3A. Progression-Free Survival of Patients Who Had Complete Metabolic Response (CMR) and Those Who Did Not Have CMR Defined by PERCIST After Stereotactic Body Radiation Therapy

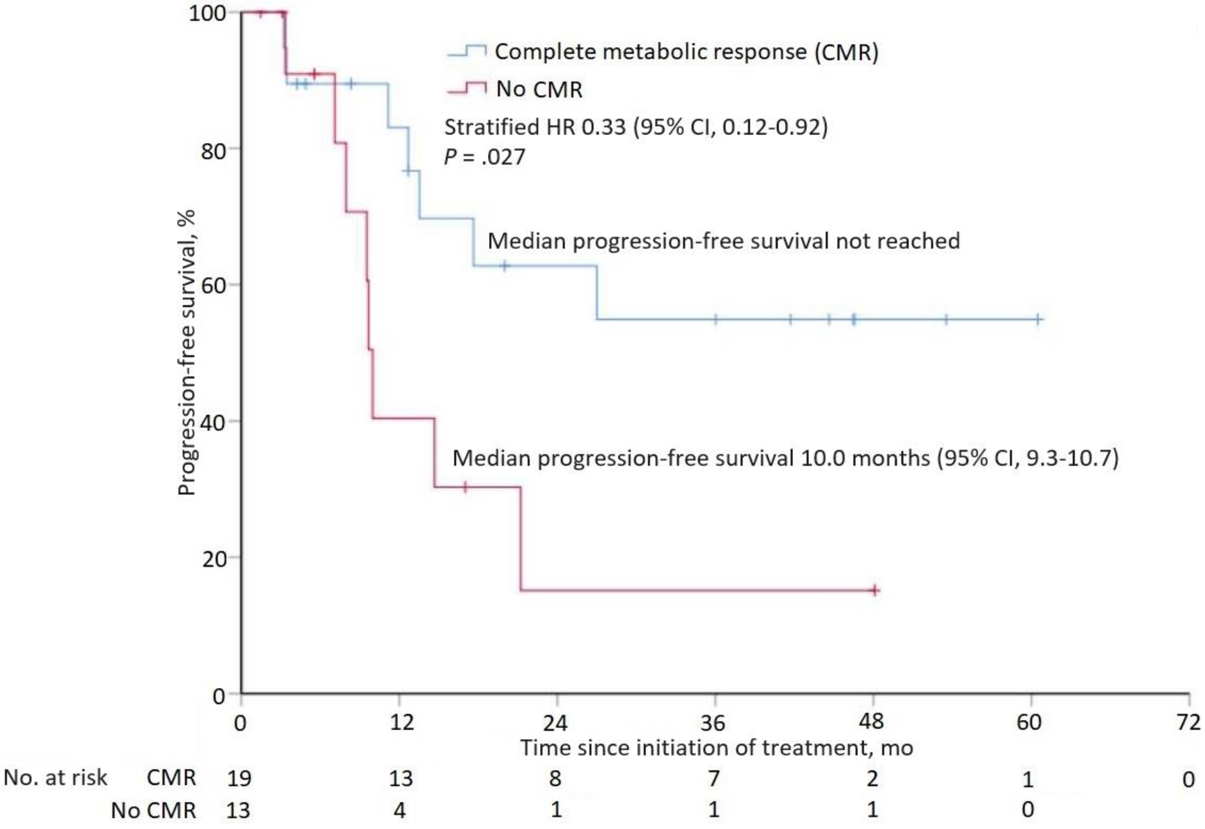

© 2024 Lee VHF et al. *JAMA Network Open.*

eFigure 3B. Overall Survival of Patients Who Had Complete Metabolic Response (CMR) and Those Who Did Not Have CMR Defined by PERCIST After Stereotactic Body Radiation Therapy

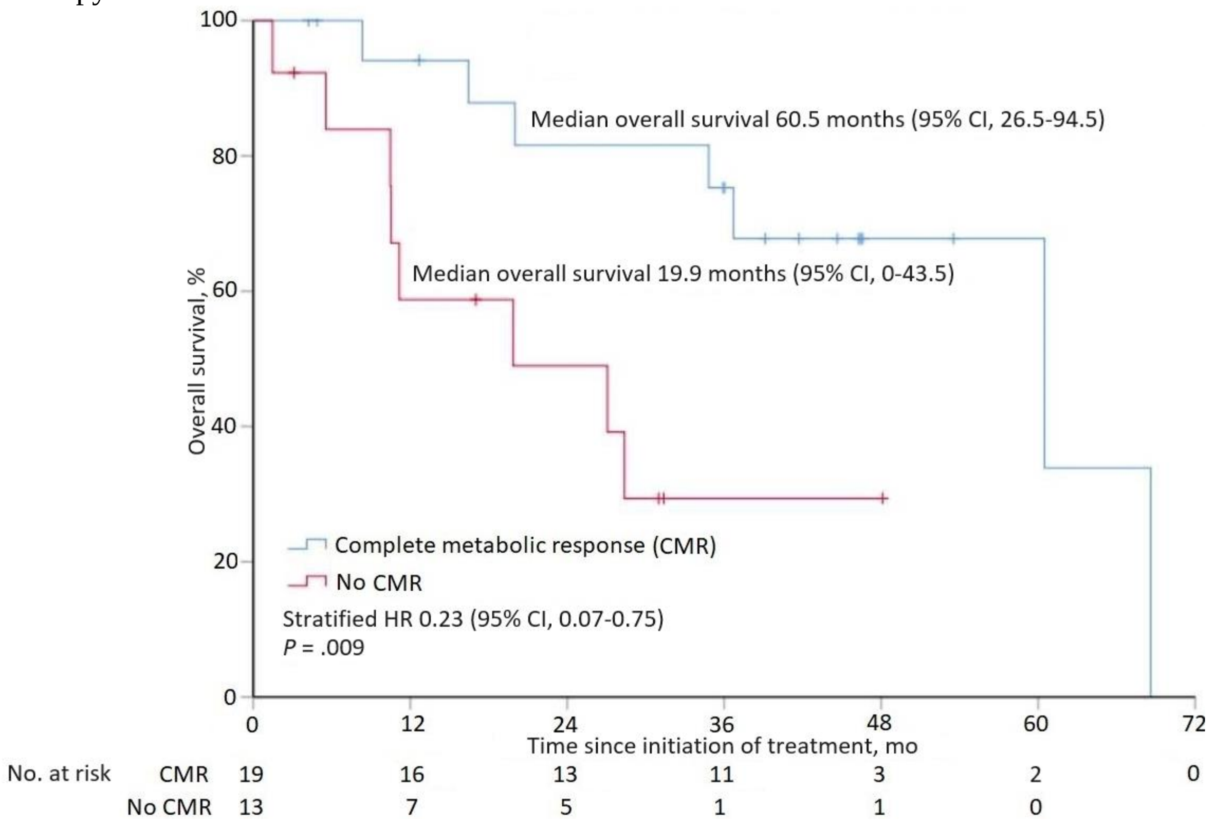

© 2024 Lee VHF et al. *JAMA Network Open.*

eFigure 4A. Progression-Free Survival of Patients Whose Hepatocellular Carcinoma Were Within the Milan Criteria and Beyond the Milan Criteria

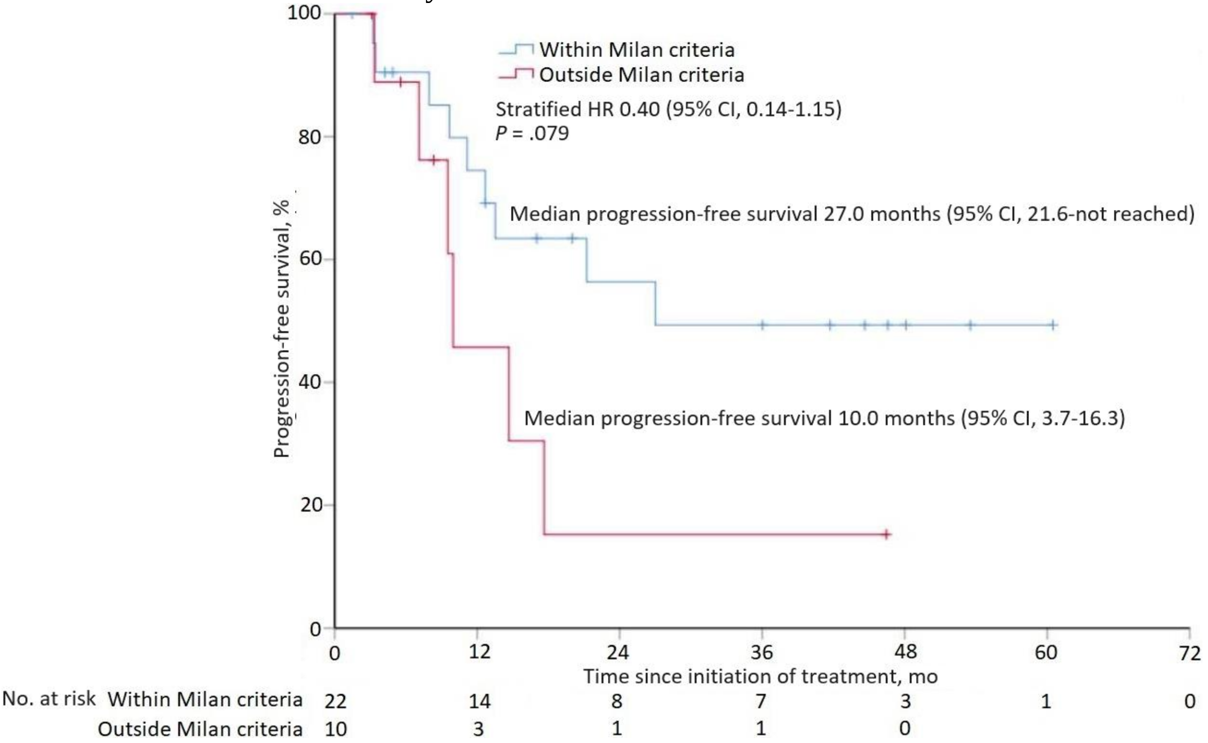

© 2024 Lee VHF et al. *JAMA Network Open.*

eFigure 4B. Overall Survival of Patients Whose Hepatocellular Carcinoma Were Within the Milan Criteria and Beyond the Milan Critieria

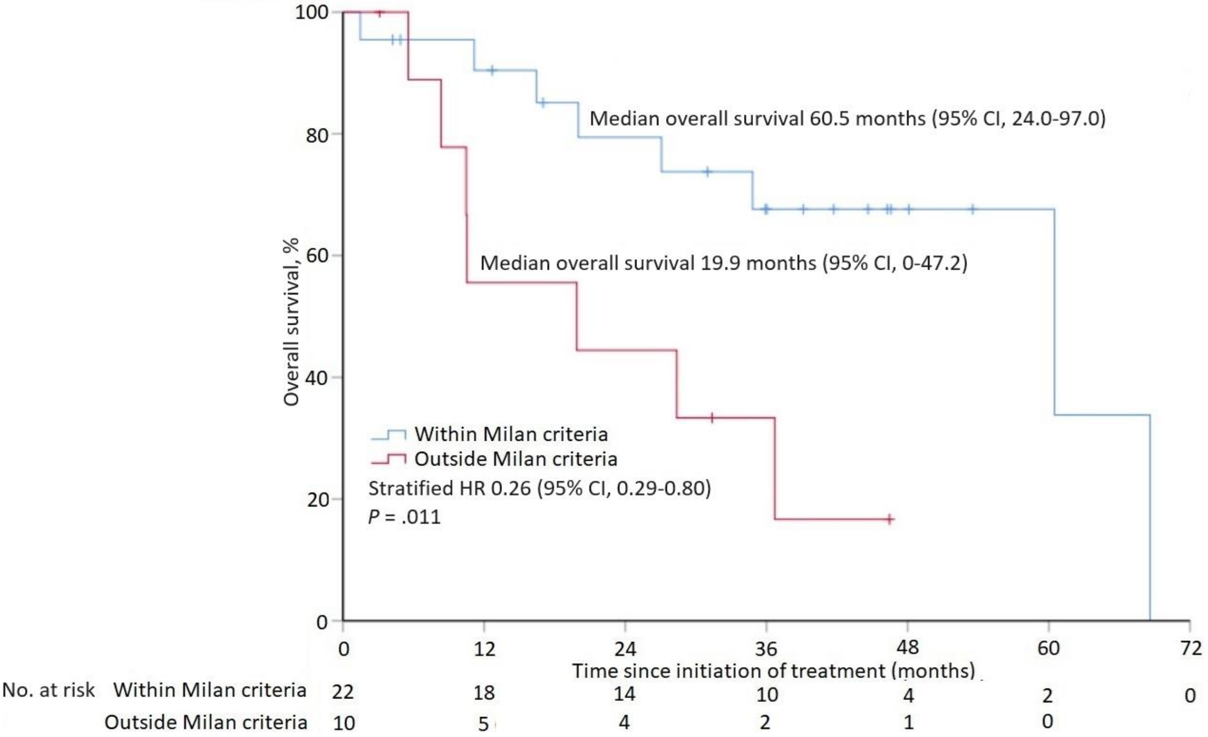

© 2024 Lee VHF et al. *JAMA Network Open.*

eFigure 5A. Progression-Free Survival of Patients Who Received and Did Not Receive Liver Transplantation After Stereotactic Body Radiation Therapy

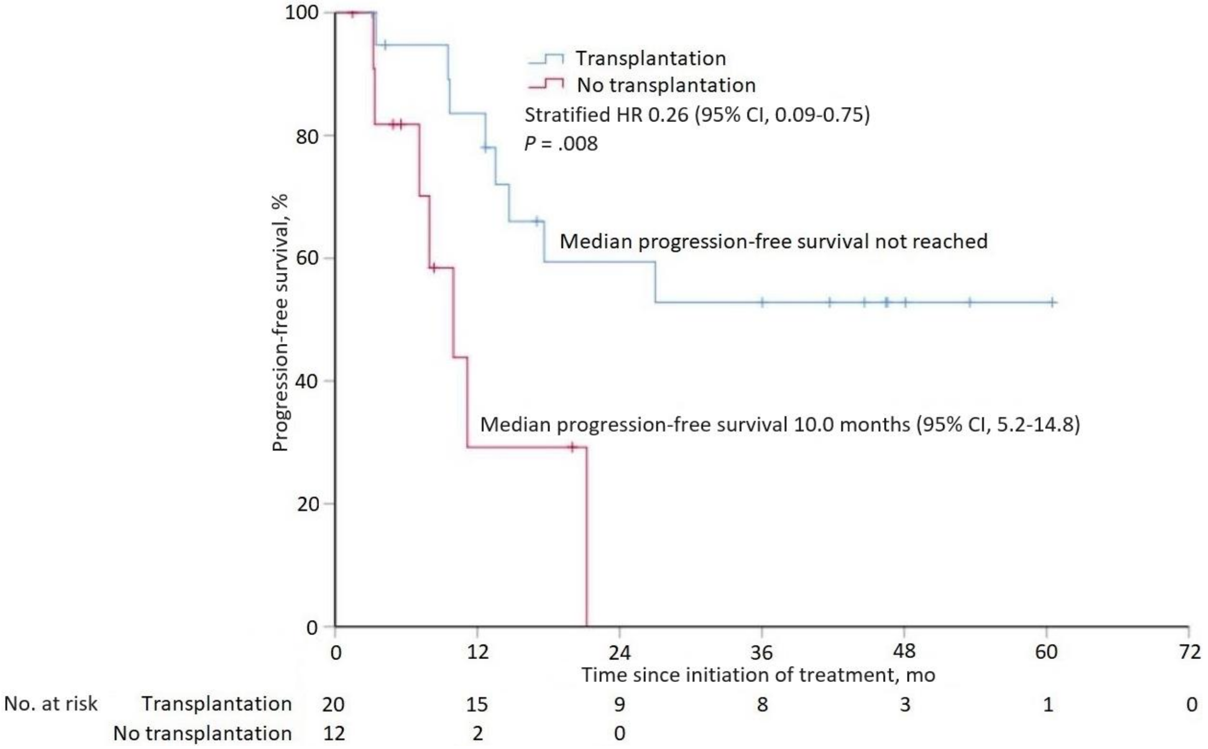

© 2024 Lee VHF et al. *JAMA Network Open.*

eFigure 5B. Overall Survival of Patients Who Received and Did Not Receive Liver Transplantation After Stereotactic Body Radiation Therapy

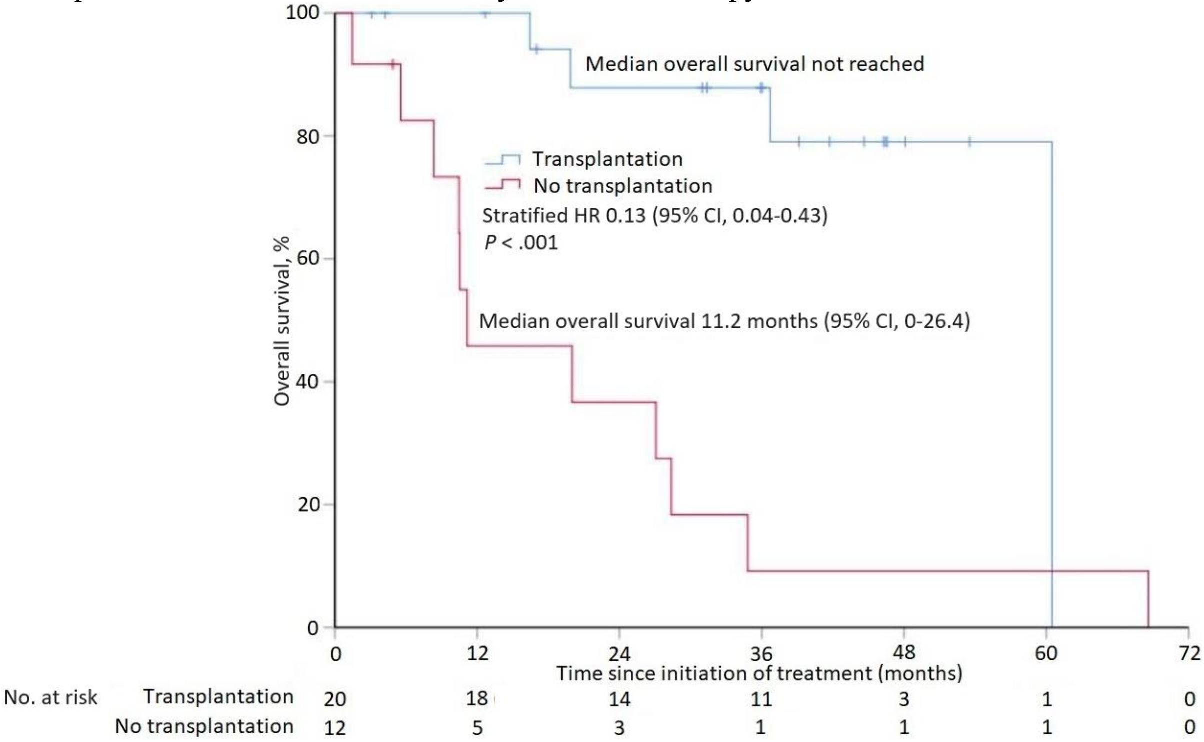

© 2024 Lee VHF et al. *JAMA Network Open.*

eTable 1. Dose Parameters of Gross Tumor Volumes and Planning Target Volumes of All Hepatocellular Carcinoma Lesions Treated With Stereotactic Body Radiation Therapy

Target volumes	Minimum (range) (Gy)	Mean (range) (Gy)	Median (range) (Gy)	Maximum (range) (Gy)
GTV	47.74 (35.49-61.63)	51.26 (39.49-63.24)	51.89 (39.57-63.28)	55.00 (41.42-64.08)
PTV	41.23 (31.12-49.94)	50.09 (38.88-59.59)	50.14 (39.00-59.37)	55.00 (41.42-65.29)
D95 of PTV	36.20	46.87	49.05	53.21

Abbreviations: D95, dose received by 95% of planning target volume; GTV, gross tumor volume; PTV, planning target volume.

© 2024 Lee VHF et al. *JAMA Network Open.*

eTable 2. Dose Parameters of All Important Organs-at-Risk of All Patients

**Organs-at-risk**	**Median dose (range) (Gy)**
Liver, mean dose (liver minus GTV)	11.58 (3.05-15.18)
D800 of normal liver, median dose	12.71 (1.22-54.24)
Common bile duct maximum (to 0.5 cc)	11.18 (0-31.02)
Common bile duct, Dmax	12.59 (0.53-37.45)
Duodenum maximum (to 0.5 cc)	11.28 (0.12-39.07)
Duodenum, Dmax	10.28 (0.15-43.21)
Gallbladder maximum (to 0.5 cc)	20.89 (1.70-46.50)
Gallbladder, Dmax	24.72 (1.89-52.51)
Kidneys, bilateral mean dose	0.59 (0.03-4.50)
Large bowel maximum (to 0.5 cc)	7.09 (0.13-47.68)
Large bowel, Dmax	7.13 (0.14-49.92)
Esophagus maximum (to 0.5 cc)	6.09 (0.98-39.10)
Esophagus, Dmax	9.11 (0.46-47.27)
Small bowel maximum (to 0.5 cc)	0.30 (0.04-14.39)
Small bowel, Dmax	0.44 (0.04-16.32)
Spinal cord maximum (to 0.5 cc)	7.21 (0.29-19.29)
Spinal cord, Dmax	8.53 (0.42-22.81)
Stomach maximum (to 0.5 cc)	11.23 (0.42-43.14)
Stomach, Dmax	12.21 (0.54-45.85)

Abbreviations: cc, cubic centimeters; D800, dose received by 800cc of normal liver; Dmax, maximum dose received by the organ-at-risk concerned.

© 2024 Lee VHF et al. *JAMA Network Open.*

eTable 3. Child-Pugh Scores Before and After Stereotactic Body Radiation Therapy (N = 32)

**Child-Pugh score**	**Baseline before SBRT, No. of patients (%)**	**Score at 1 month after SBRT (%)**	***P***	**Score at 2 months after SBRT (%)**	***P***	**Score at 3 months after SBRT (%)**	***P***	**Score at 6 months after SBRT (%)**	***P***
5	8 (25.0)	8 (25.0)	.109	7 (21.9)	.083	5 (15.6)	.044	7 (21.9)	.262
6	12 (37.5)	7 (21.9)		7 (21.9)		8 (25.0)		10 (31.3)	
7	6 (18.7)	9 (28.1)		9 (28.1)		10 (31.3)		8 (25.0)	
8	6 (18.8)	7 (21.9)		8 (25.0)		8 (25.0)		5 (15.6)	
9	0 (0)	0 (0)		1 (3.1)		0 (0)		0 (0)	
10	0 (0)	1 (3.1)		0 (0)		0 (0)		1 (3.1)	
Total	32 (100)	32 (100)		32 (100)		32 (100)		32 (100)	

Abbreviation: SBRT, stereotactic body radiation therapy.

© 2024 Lee VHF et al. *JAMA Network Open.*

eTable 4. Change in Child-Pugh Scores After Stereotactic Body Radiation Therapy (N = 32)

Change in Child-Pugh score after SBRT	1 month after SBRT, No. of patients (%)	2 months after SBRT, No. of patients (%)	3 months after SBRT, No. of patients (%)	6 months after SBRT, No. of patients (%)
-3[a]	0 (0)	0 (0)	0 (0)	1 (3.1)
-2	1 (3.1)	1 (3.1)	1 (3.1)	1 (3.1)
-1	5 (15.6)	5 (15.6)	4 (12.5)	5 (15.6)
0	13 (40.6)	13 (40.6)	13 (40.6)	11 (34.4)
+1	10 (31.3)	9 (28.1)	8 (25.0)	10 (31.3)
+2	3 (9.4)	3 (9.4)	4 (12.5)	2 (6.3)
+3[b]	0 (0)	1 (3.1)	1 (3.1)	1 (3.1)
Total	32 (100)	32 (100)	32 (100)	32 (100)

[a] This patient had a baseline Child-Pugh score 8 which dropped to 5 at 6 months after stereotactic body radiation therapy.

[b] This patient with a baseline Child-Pugh score 8 was found to have progressive disease with numerous and disseminated intrahepatic metastases at 3 months after stereotactic body radiation.

Abbreviation: SBRT, stereotactic body radiation therapy.

© 2024 Lee VHF et al. *JAMA Network Open.*

eTable 5. Number of Patients Whose Pretreatment Parameters Are Beyond RTOG 1112 Trial Eligibility Criteria

	**Minimum**	**Maximum**	**Interquartile range**	**No. of patients beyond RTOG 1112 trial eligibility criteria**
Albumin (g/liter)	26	26	26-26	2
Platelet ($\times 10^9$/liter)	20	69	30-51	20
Bilirubin in mg/dl (μmol/liter)	2.05 (35)	3.27 (56)	2.11–3.10 (36-53)	10
Total				23[a]

[a] Altogether 9 patients had 2 or more parameters beyond the RTOG 1112 trial eligibility criteria.

© 2024 Lee VHF et al. *JAMA Network Open.*

eTable 6. Number of Patients Whose Dosimetric Parameters of Organs-at-Risk in Their SBRT Plans Beyond RTOG 1112 Trial Acceptance Criteria

	**Minimum (Gy)**	**Maximum (Gy)**	**Interquartile range (Gy)**	**No. of patients beyond RTOG 1112 trial eligibility criteria**
Esophagus (to 0.5 cc)	39.10	39.10	39.10	1
Stomach (to 0.5 cc)	34.17	43.14	36.69-39.92	4
Duodenum (to 0.5 cc)	39.07	39.07	39.07	1
Large bowel (to 0.5 cc)	39.14-47.68	47.68	41.86-44.12	4
Total				7[a]

[a] Altogether 3 patients had 2 or more dosimetric parameters exceeding the dose tolerance of organs-at-risk in RTOG 1112 trial acceptance criteria.

Abbreviation: SBRT, stereotactic body radiation therapy.

© 2024 Lee VHF et al. *JAMA Network Open.*
