# Supplementary material for: Stereotactic Body Radiotherapy and Liver Transplant for Liver Cancer: A Nonrandomized Controlled Trial
Source: JAMA Netw Open. 2024 Jun 10;7(6):e2415998. doi: 10.1001/jamanetworkopen.2024.15998 (PMC11165380; doi:10.1001/jamanetworkopen.2024.15998)
Supplement: Supplement 3. — Data Sharing Statement [file jamanetwopen-e2415998-s003.pdf]

Data Sharing Statement

Lee. Stereotactic Body Radiotherapy and Liver Transplant for Liver Cancer. *JAMA Netw Open*. Published June 10, 2024. doi:10.1001/jamanetworkopen.2024.15998

Data
Data available: Yes
Data types: Deidentified participant data
How to access data: The data sets, including the redacted study protocol, redacted statistical analysis plan, and individual participant data supporting the results reported in this article, will be made available within three months from initial request, to researchers who provide a methodologically sound proposal. The data will be provided after de-identification, in compliance with applicable privacy laws, data protection, and requirements for consent and anonymization.
When available: With publication

Supporting Documents
Document types: None

Additional Information
Who can access the data: The data will be made available within three months from initial request, to researchers who provide a methodologically sound proposal. The data will be provided after de-identification, in compliance with applicable privacy laws, data protection, and requirements for consent and anonymization.
Types of analyses: The data will be made available for pooled analysis or meta-analysis.
Mechanisms of data availability: The data will be made available following a signed data access agreement.
Any additional restrictions: Inapplicable
